# Pathophysiological role of microRNA-29 in pancreatic cancer stroma

**DOI:** 10.1038/srep11450

**Published:** 2015-06-22

**Authors:** Jason J. Kwon, Sarah C. Nabinger, Zachary Vega, Smiti Snigdha Sahu, Ravi K. Alluri, Zahi Abdul-Sater, Zhangsheng Yu, Jesse Gore, Grzegorz Nalepa, Romil Saxena, Murray Korc, Janaiah Kota

**Affiliations:** 1 Department of Medical and Molecular Genetics, Indiana University School of Medicine (IUSM), Indianapolis, IN, USA; 2 Wabash College, Crawfordsville, IN, USA; 3 Department of Pathology, IUSM, Indianapolis, IN, USA; 4 Department of Biochemistry and Molecular Biology, IUSM, Indianapolis, IN, USA; 5 Department of Biostatistics, IUSM, Indianapolis, IN, USA; 6 Department of Medicine, IUSM, Indianapolis, IN, USA; 7 The Melvin and Bren Simon Cancer Center, IUSM, Indianapolis, IN, USA; 8 Center for Pancreatic Cancer Research, Indiana University and Purdue University-Indianapolis (IUPUI), Indianapolis, IN, USA; 9 Department of Pediatrics, Herman B Wells Center for Pediatric Research, IUSM, Indianapolis, IN, USA; 10 Division of Pediatric Hematology-Oncology, Bone Marrow Failure Program, IUSM, Indianapolis, IN, USA

## Abstract

Dense fibrotic stroma associated with pancreatic ductal adenocarcinoma (PDAC) is a major obstacle for drug delivery to the tumor bed and plays a crucial role in pancreatic cancer progression. Current, anti-stromal therapies have failed to improve tumor response to chemotherapy and patient survival. Furthermore, recent studies show that stroma impedes tumor progression, and its complete ablation accelerates PDAC progression. In an effort to understand the molecular mechanisms associated with tumor-stromal interactions, using *in vitro* and *in vivo* models and PDAC patient biopsies, we show that the loss of miR-29 is a common phenomenon of activated pancreatic stellate cells (PSCs)/fibroblasts, the major stromal cells responsible for fibrotic stromal reaction. Loss of miR-29 is correlated with a significant increase in extracellular matrix (ECM) deposition, a major component in PDAC stroma. Our *in vitro* miR-29 gain/loss-of-function studies document the role of miR-29 in PSC-mediated ECM stromal protein accumulation. Overexpression of miR-29 in activated stellate cells reduced stromal deposition, cancer cell viability, and cancer growth in co-culture. Furthermore, the loss of miR-29 in TGF-β1 activated PSCs is SMAD3 dependent. These results provide insights into the mechanistic role of miR-29 in PDAC stroma and its potential use as a therapeutic agent to target PDAC.

PDAC is the most lethal human cancer, with a 5-year survival rate of 6%^1^ and is projected to be the second leading cause of cancer related death by 2030^2^. Dense fibrotic stroma associated with PDAC tumors is a major obstacle for drug delivery to the tumor bed^3^,^4^ and plays a crucial role in pancreatic cancer progression^5^,^6^,^7^. Current anti-stromal therapies have failed to improve tumor response to chemotherapy and patient survival^8^, and they are often associated with toxicity^9^. Furthermore, recent studies show that the stroma impedes tumor growth, and its complete inhibition accelerated PDAC progression^10^,^11^. However, reprogramming the stromal reaction through modulation of a key transcriptional regulator in stromal cells suppressed the stromal reaction and increased tumor response to chemotherapy and survival^12^. These studies reinforce the critical need to understand the molecular mechanisms associated with tumor-stromal interactions for developing effective therapeutic strategies to reduce PDAC mortality.

Stroma associated with PDAC tumors is comprised of activated stellate cells/fibroblasts, immune cells, extracellular matrix, and pro-inflammatory cytokines/growth factors^13^ (Supp Fig. 1). A dynamic interaction between tumor-stromal cells and the extracellular matrix has been shown to play a critical role in PDAC progression^13^. A growing body of evidence suggests that pancreatic stellate cells (PSCs) are the major stromal cells responsible for fibrotic stroma. In normal pancreas, PSCs are located in periacinar spaces and remain quiescent. In response to pancreatic injury or carcinogenesis, PSCs are activated by a number of pro-inflammatory growth factors and cytokines, including transforming growth factor β1 (TGF-β1) released by cancer cells, injured acinar cells (paracrine), and activated PSCs (autocrine). TGF-β1 serves as a key activator of quiescent PSCs, which is highly expressed in PDAC tumors and is known to play an important role in the fibrotic process associated with pancreatitis and PDAC pathogenesis^14^,^15^. Activated PSCs are transformed into a myofibroblast-like phenotype and secrete excessive amounts of ECM proteins such as collagen, laminin, and fibronectin, a prominent component of PDAC stroma^16^. Extensive ECM deposition causes hypovascularity in PDAC tumors and attenuates drug delivery to the peritumoral milieu^17^. Furthermore, PSCs closely interact with cancer cells and the ECM to promote primary tumor growth and distant metastasis^6^,^18^,^19^,^20^. However, the molecular mechanisms associated with stellate-cancer interactions remain poorly understood.

MicroRNAs (miRNAs) are conserved, non-coding RNAs that regulate eukaryotic gene expression and are critical in maintaining cellular homeostasis^21^. A single miRNA regulates hundreds of genes, often targeting multiple components of complex intracellular networks^22^. Thus, misregulation of an individual miRNA can have a profound impact on cellular physiology that leads to disease(s)^23^. miR-29 is known to play a paramount role in the fibrotic process of several organs^24^,^25^,^26^,^27^,^28^ and provides crucial functions downstream of pro-fibrotic signaling pathways such as TGF-β1^29^. Numerous functional studies have documented the anti-fibrotic activity of miR-29 in different tissues^30^, and its restored expression reduced fibrosis by targeting ECM proteins^31^, such as collagen and laminin. Additionally, a growing body of evidence implicates the role of miR-29 in cancer pathogenesis^32^ and in the tumor microenvironment^33^. The role of miR-29 in pancreatic fibrosis and PDAC tumor-stromal interactions however, has yet to be delineated.

Here in this study, we show that the loss of miR-29a and b is a common feature of activated PSCs and fibroblasts and is associated with a significant increase in stromal ECM deposition *in vitro* and *in vivo*. Our *in vitro* miR-29 gain/loss-of-function studies directly document the role of miR-29 in PSC-mediated stromal accumulation. In addition, overexpression of miR-29a or b in PSCs decreased stroma deposition, cancer cell viability, and colony growth when co-cultured with pancreatic cancer cells. Our findings show that miR-29 plays a critical role in regulating tumor stromal deposition and cancer growth, thus raising the possibility that modulation of miR-29 expression in PSCs may be therapeutically beneficial to normalize the reactive stroma and enhance the efficacy of chemotherapy to target PDAC.

## Results

### TGF-β1 activates PSCs, downregulates miR-29, and increases ECM protein expression

**Figure 1:**
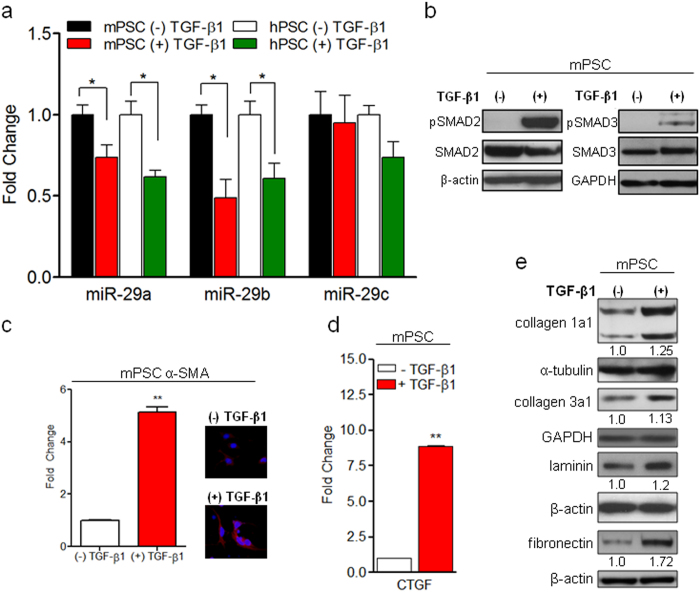
TGF-β1-mediated activation of PSCs leads to downregulation of miR-29 and increases ECM protein expression. Mouse PSCs (mPSCs) and human PSCs (hPSCs) were serum starved for 24 hours and stimulated with 10 ng/ml of TGF-β1 for 24 hours. Total RNA or protein were isolated for qPCR and western blot analysis respectively. (**a**) qPCR analysis of miR-29 family members in nascent and TGF-β1 stimulated mPSCs and hPSCs. (**b**) Activation of pSMAD2/3 in TGF-β1 stimulated mPSCs. Serum starved mPSCs were treated with TGF-β1 for 6 hours and subjected to western blot analysis for pSMAD2/3 and total SMAD2/3 expression. β-actin and GAPDH were used as loading controls. (**c**) qPCR and immunofluorescence analysis of PSC activation marker, alpha smooth muscle actin (α-SMA) in TGF-β1 stimulated mPSCs. Representative images are shown, α-SMA (red) and nuclear stain, DAPI (blue). (**d**) qPCR analysis of TGF-β1 responsive transcriptional gene target, connective tissue growth factor (CTGF) in nascent and TGF-β1 stimulated mPSCs. (**e**) Western blot analysis of extracellular matrix proteins: collagen 1alpha1 (collagen 1a1), collagen 3alpha1 (collagen 3a1), laminin gamma-1 (laminin), and fibronectin in nascent and TGF-β1 activated mPSCs. α-tubulin, or GAPDH, or β-actin were used as loading controls. Relative quantification of band intensities, normalized to loading controls, are shown below respective blots. Experiments were repeated 3-4 times and representative data are shown. Data is presented as the mean + standard error of the mean (SEM); n = 3; p-values determined by t-test, *p < 0.05, **p < 0.01.

In PDAC, activated PSCs are the major stromal cells responsible for fibrotic stroma. In an effort to evaluate the role of miR-29, an anti-fibrotic miRNA, in PSC-mediated stromal deposition, we challenged immortalized mouse PSCs and primary human PSCs with TGF-β1, a proinflammatory growth factor that is associated with PDAC pathogenesis^14^ and that is known to activate quiescent PSCs^16^. Subsequently, we examined miR-29 family (-29a, -29b & -29c) expression in TGF-β1 activated mouse and human PSCs by qPCR analysis. Both mouse and human PSCs stimulated with TGF-β1 showed a significant loss of miR-29a and miR-29b compared to nascent cells, with no significant difference in miR-29c (Fig. 1a and Supp Fig. 2). TGF-β1 bound TGF-β receptors (type I and type II) form an activated heterotetramer with serine/threonine kinase activity which phosphorylates downstream transcription factors, Smad2 and Smad3 proteins^34^. Upon phosphorylation and activation, pSMAD2/3 form a heterogeneous complex with SMAD4, translocates into the nucleus, and directly regulates target gene expression. To verify the activation of TGF-β1 signaling in PSCs, we examined the levels of its downstream effector molecules, pSMAD2/3. PSCs treated with TGF-β1 showed a significant increase in pSMAD2/3 levels with no difference in total SMAD2/3 expression in both mouse and human PSCs (Fig. 1b and Supp Fig. 3). To further confirm TGF-β1-mediated activation of PSCs and TGF-β1 signaling, we evaluated the expression of α-SMA, a myofibroblast/PSC activation marker^16^, and connective tissue growth factor (CTGF), a transcriptional target and mediator for the pro-fibrotic effects of TGF-β1. We observed a decrease in miR-29a and miR-29b expression (Fig. 1a) with a concurrent increase in α-SMA (Fig. 1c) and CTGF (Fig. 1d) expression. Upregulation of TGF-β1 and CTGF has been well documented in human PDAC and chronic pancreatitis, a major risk factor for PDAC^14^,^15^,^35^, and causes an increase in the accumulation of stromal ECM proteins: collagen, laminin, and fibronectin^13^. Furthermore, collagen and laminin are direct targets of miR-29^31^, and fibronectin is an indirect target of miR-29^26^. We observed their significant increase in PSCs treated with TGF-β1 in conjunction with a loss of miR-29a and miR-29b (Fig. 1e and Supp Fig. 4). Consistently, overexpression of miR-29 has been shown to suppress the fibrosis of various organs, including heart^31^, liver^24^, lung^26^, kidney^25^, and muscle^27^ by reducing ECM deposition.

### TGF-β1 downregulates miR-29 in human fibroblasts and cancer associated fibroblasts

**Figure 2:**
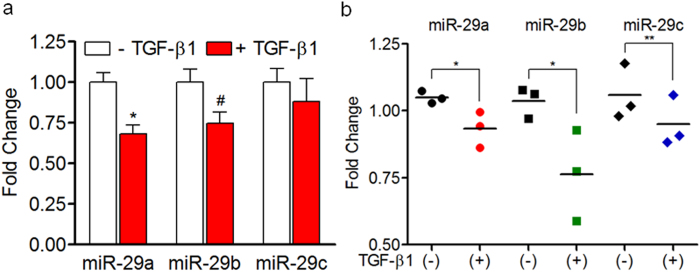
TGF-β1 downregulates miR-29 in human fibroblasts and cancer associated fibroblasts. Primary human pancreatic fibroblasts or cancer associated fibroblasts (CAFs) isolated from human PDAC tumors were stimulated with 10 ng/ml of TGF-β1 for 24 hours, and total RNA was isolated for miR-29 expression analysis. (**a**) qPCR analysis of miR-29 family members in TGF-β1 activated primary human pancreatic fibroblasts or (**b**) PDAC CAFs (n = 3). Data is presented as the mean + SEM; n = 3; p-values determined by t-test, *p < 0.05, ^#^p < 0.08, **p < 0.01. Experiments were repeated three times and representative data are shown.

In addition to PSCs, pancreatic fibroblasts are also known to play a role in the stromal reaction associated with PDAC tumors^5^. To understand whether the loss of miR-29 is common in TGF-β1 activation, we treated normal primary human pancreatic fibroblasts with TGF-β1 and quantified miR-29 expression levels. Similar to PSCs, TGF-β1-activated primary fibroblasts had decreased miR-29a expression compared to nascent cells (Fig. 2a), suggesting that the loss of miR-29a is consistent in both activated PSCs and fibroblasts. During PDAC initiation and progression, normal pancreatic fibroblasts convert into an activated state and are known as cancer associated fibroblasts (CAFs). In addition to PSCs, CAFs have been shown to play a role in PDAC stromal deposition^5^, initiation^36^, progression, and metastasis^37^. To determine whether TGF-β1 dependent loss of miR-29 is a common phenomenon in CAFs, we examined the effects of TGF-β1 treatment on miR-29 expression in primary CAFs isolated from PDAC patients. Similarly, CAFs challenged with TGF-β1 showed a significant decrease in the expression of miR-29 family members (Fig. 2b), suggesting that the loss of miR-29 function is a common phenomenon of activated stromal cells associated with fibrotic stromal deposition.

### Activation of *Kras* ^G12D^ in the pancreas leads to loss of miR-29 and increased collagen deposition

**Figure 3:**
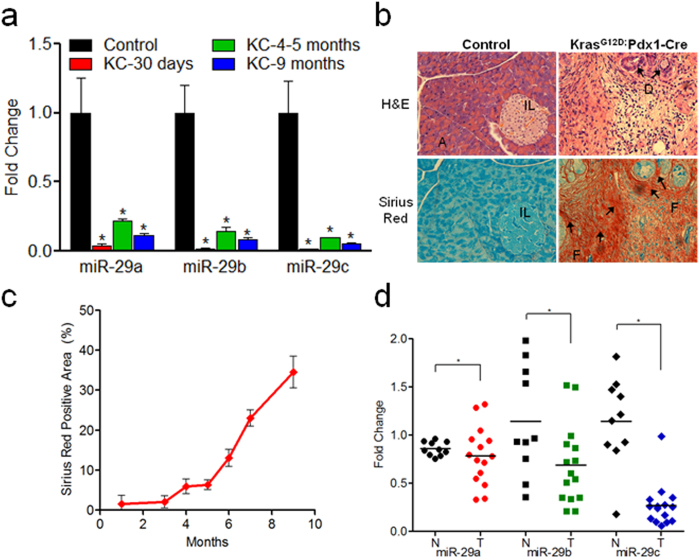
Global loss of miR-29 is a common phenomenon of *Kras*^G12D^ activated murine pancreata and human PDAC tumors with increased collagen deposition. (**a**) miR-29 expression levels in LSL-*Kras*^*G12D*^; *Pdx1-Cre* (KC), PDAC mouse model. Total RNA was isolated from formalin fixed paraffin embedded (FFPE) pancreatic tissue sections of KC or C57BL/6 control mice (n = 3–5 animals/time point) and subjected to miR-29 expression analysis via qPCR. (**b**) Representative H&E and Sirius Red stained pancreatic sections from C57BL/6 control and KC mice (9 months). Acinar cells (A), Islets of Langerhans (IL), pancreatic ducts (D), and prominent fibrosis (F) are demonstrated in the KC animals. (**c**) Quantification of collagen in KC animals from 1–9 months of age. ImageJ was used to quantify Sirius Red positive collagen. Data is presented as the mean ± SEM; n = 3–5 animals/time point; (**d**) miR-29 expression analysis in PDAC patient tumors. Total RNA was isolated from FFPE sections of normal controls (n = 10) (N; solid black) or PDAC patient tumors (n = 15) with 35–80% stroma (T; red, green, or blue), and miR-29 expression was analyzed by qPCR. miR-29 family members are represented by circles (miR-29a), squares (miR-29b), and diamonds (miR-29c). For each group, the mean expression of miR-29 family members is indicated as horizontal lines. p-values determined by t-test, *p < 0.05.

Activating mutations in the proto-oncogene *KRAS* are common in 90%–95% of PDAC patients, and *Kras*^*G12D*^ is the most frequently found genetic aberration^38^. In order to explore the effects of *Kras*^*G12D*^on miR-29 expression, we collected pancreata from a well-characterized PDAC mouse model, *LSL Kras*^*G12D*^; *Pdx1-Cre* (KC)^39^, at 1–9 months of age and examined global pancreatic miR-29 expression and collagen/connective tissue deposition. We observed a significant loss of miR-29 expression in KC mice compared to C57BL/6 controls (Fig. 3a) in conjunction with a significant increase in pancreatic fibrosis/collagen estimation by gross histopathological examination (H&E and Sirius Red stain) (Fig. 3b) and quantification of Sirius Red positive collagen (Fig. 3c).

### Global loss of miR-29 expression in human PDAC tumors

To determine the miR-29 expression patterns in human PDAC tumors and establish its clinical relevance, we examined miR-29 expression in PDAC patient biopsies with 35–80% tumor stroma, assessed via Sirius Red staining, and compared them to normal patient control samples. Similar to KC mice, we observed a significant decrease in all miR-29 family members in PDAC tumor samples compared to normal patient controls (Fig. 3d). Furthermore, we conducted H&E and Sirius Red staining and observed a corresponding increase in fibrosis in PDAC tumor biopsies by both gross histopathological examination (Supp Fig. 5a) and quantification of Sirius Red staining (Supp Fig. 5b).

### PSC and epithelial cell specific miR-29 loss of expression in KC mice and human PDAC tumors

**Figure 4:**
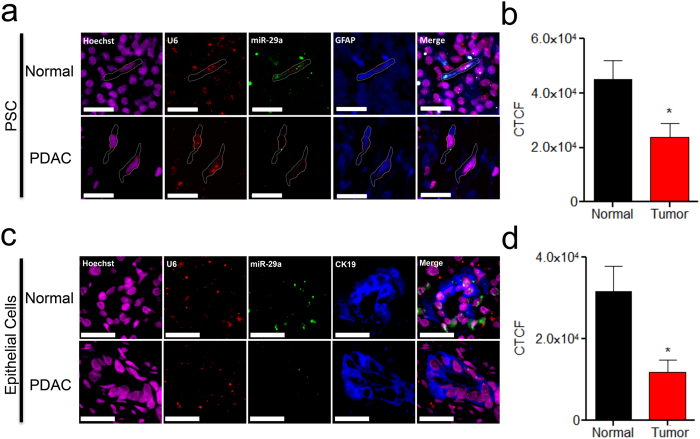
PSC and epithelial cell specific miR-29 loss of expression in human PDAC tumors. (a, c) *In situ* hybridization of miR-29 in PSCs (a) and epithelial cells (c) of normal control and PDAC patient tumors. FFPE pancreatic tissue sections from normal control and PDAC patients (n = 4/group) were subjected to miR-29a *in situ* hybridization. Representative images are presented as a single channel or merged (scale bar is 5 μm, 20X magnification). Hoechst nuclear stain (magenta), U6 (red), miR-29a (green), and GFAP-positive PSCs or CK19-positive epithelial cells (blue). (b, d) Corrected total cell fluorescence (CTCF) of miR-29a in PDAC tumors (n = 4) compared to control patients (n = 4) was calculated for each patient averaging six or more randomly selected GFAP-positive PSCs (b) or CK19-positive epithelial cells (d) using ImageJ analysis. Data represents mean + SEM. Statistics were generated using t-test, *p < 0.05.

As we observed global loss of miR-29 in both human PDAC tumors and KC mice, to examine PSC and epithelial cell specific miR-29 expression patterns in PDAC tumors and KC mice pancreata, we performed *in situ* hybridization of miR-29a, the most abundantly expressed miR-29 family member in PSCs, epithelial cells, and the pancreas (Supp Fig. 6). To assess PSC specific miR-29 expression, we co-stained PDAC tumors and KC mice pancreata with miR-29a and glial fibrillary acidic protein (GFAP), a cell marker of both active and inactive PSCs/fibroblasts^40^. Consistent with PSCs/fibroblasts challenged with TGF-β1, GFAP positive cells of PDAC tumors displayed a significant loss of miR-29a compared to normal patient control pancreata (Fig. 4a,b). Similarly, KC mice displayed significant loss of miR-29a in GFAP-positive cells compared to control mice at early (2–4 months) and late ages (9–10 months) (Supp Fig. 7).

To examine epithelial cell specific miR-29 expression patterns, we co-stained human PDAC tumors and KC mice pancreata with miR-29a, and Cytokeratin-19 (CK19), a cell marker specific to epithelial cells^41^. Similar to PSCs, CK19-positive epithelial cells of PDAC tumors (Fig. 4c,d) and KC mice (Supp Fig. 8) showed a significant loss of miR-29 expression compared to normal controls, indicating that the global loss of miR-29 may be attributed by both stromal PSCs and epithelial cells.

### Physiological role of miR-29 in PSC-mediated stromal ECM protein accumulation

**Figure 5:**
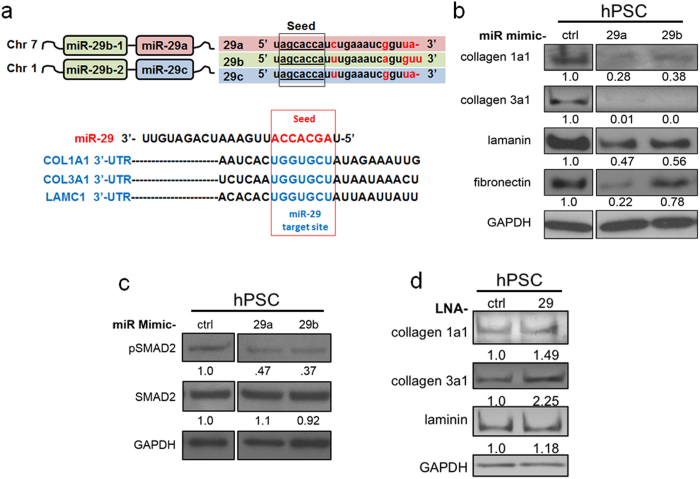
Physiological role of miR-29 in PSC-mediated stromal ECM protein accumulation. For miR-29 gain and loss-of-function studies, serum starved and TGF-β1 (10 ng/ml, 24 hours) treated PSCs were transfected with synthetic miR-29 mimics or miR-29 locked nucleic acids (LNAs), a miR-29 family inhibitor respectively. Subsequently, the effects of miR-29 gain and loss-of-function on extracellular matrix (EMC) proteins was evaluated by western blot analysis. (**a**) Schematic representation of the miR-29 family members, and 3′-untranslated region (UTR) binding sites of miR-29 ECM targets. miR-29 encoding loci are located on human chromosome 7 (miR-29a/miR-29b-1) and chromosome 1 (miR-29b-2/miR-29c). All three miR-29 family members (miR-29a, miR-29b, and miR-29c) have identical seed sequences. miR-29 binding sites in the 3′-UTR of ECM protein transcripts encoding collagen 1a1 (COL1A1), collagen 3a1 (COL3A1), and laminin gamma-1 (LAMC1) are depicted. (**b**) Western blot analysis of ECM proteins in miR-29 gain-of-function studies of hPSCs. TGF-β1 stimulated hPSCs were transfected with 20 nM mimic control (ctrl), mimic-29a (29a), or mimic-29b (29b). 24 hours post-transfection, total proteins were harvested and subjected to western blot analysis of ECM proteins (collagen 1a1, collagen 3a1, laminin, and fibronectin). (**c**) Western blot analysis of pSMAD2 in miR-29 overexpressed hPSCs. Serum starved and TGF-β1 treated hPSCs were transfected with miR-29 (20 nM) or control mimics (20 nM). 24 hours post-transfection, protein was harvested and subjected to western blot analysis for pSMAD2 and SMAD2. (**d**) Western blot analysis of ECM proteins (collagen 1a1, collagen 3a1, and laminin) in miR-29 loss-of-function studies of hPSCs. TGF-β1 treated hPSCs were transfected with 50 nM of LNA-29 or LNA-control. 24 hours post-transfection, protein was harvested and subjected to western blot analysis. Relative quantification of band intensities, normalized to GAPDH loading control, are shown below respective blots. Each experiment was repeated three times and representative data are presented.

Since the loss of miR-29a/b was commonly observed in PSCs/fibroblasts challenged with TGF-β1, in *Kras*^*G12D*^ expressing murine pancreata, and in human PDAC biopsies, we performed *in vitro* gain/loss-of-function studies to determine the physiological role of miR-29 in PSC-mediated stromal protein expression using synthetic miR-29 mimics and miR-29 locked nucleic acids (LNAs), a miR-29 family inhibitor. To test the effect of miR-29 gain-of-function on PSC-mediated stromal accumulation, we transfected mouse and human PSCs with control, miR-29a, or b mimics, before (data not shown) and after TGF-β1 treatment, and examined ECM protein levels. We confirmed the overexpression of miR-29a and miR-29b in both mouse and human PSCs transfected with synthetic miR-29a/b mimics (Supp Fig. 9). As expected, we observed a significantly lower expression of direct miR-29 ECM protein targets (Fig. 5a) in PSCs transfected with miR-29 mimics compared to cells transfected with control mimic. Interestingly, in miR-29 overexpressed PSCs, we also saw a decrease in fibronectin, a major ECM protein in PDAC stroma and a known indirect target of miR-29^26^ (Fig. 5b and Supp Fig. 10).

In order to better understand the underlying mechanism for miR-29-mediated fibronectin repression in TGF-β1 activated PSCs, we investigated the effect of miR-29 on TGF-β1 signaling. We observed a decrease in pSMAD2 levels in PSCs transfected with miR-29a or miR-29b compared to control mimic (Fig. 5c and Supp Fig. 11), suggesting an indirect effect of miR-29 on fibronectin suppression.

For miR-29 loss-of-function studies, we transfected TGF-β1 activated PSCs with control or miR-29 LNAs and assessed the effects on stromal protein accumulation. We confirmed the knockdown of all three miR-29 family members in PSCs transfected with miR-29 LNAs (Supp Fig. 12). Inhibition of miR-29 by LNAs led to a further increase in accumulation of ECM proteins (Fig. 5d and Supp Fig. 13).

### Ectopic expression of miR-29 in PSCs reduces stromal deposition, cancer cell viability, and colony growth in co-culture

**Figure 6:**
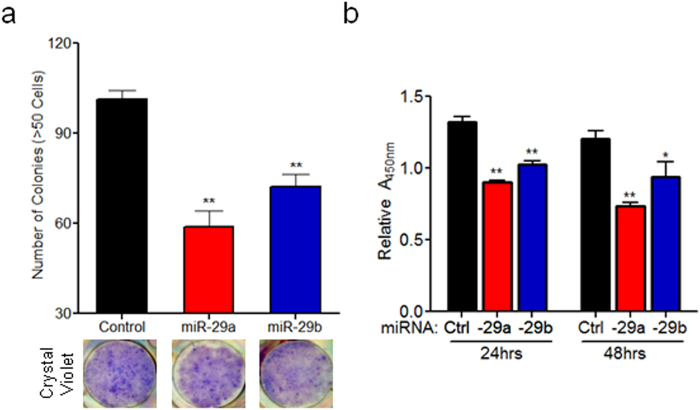
Ectopic expression of miR-29 in PSCs reduces cancer cells viability and cancer colony growth in co-culture. (**a**) Effect of miR-29 overexpression in PSCs on cancer colony growth in direct co-cultures. mPSCs transfected with 20 nM mimic control, mimic-29a or 29b were plated simultaneously with 100 pancreatic cancer cells (Panc-1) in a 6-well plate. Co-cultures were allowed to grow for 10 days, fixed, and stained with crystal violet fixing solution to stain cancer cells as previously described^64^ with few modifications. Cancer colonies greater than 50 cells were counted under phase contrast microscopy. Representative images of co-cultures stained with crystal violet are shown. (**b**) Conditioned media of PSCs expressing miR-29 show decreased effect on pancreatic cell viability. Conditioned media from mPSCs transfected with control, miR-29a, or miR-29b mimics was applied to pancreatic cancer cells (Panc-1) in a 96-well plate and viability was measured 24 and 48 hours post-treatment using the Cell Counting Kit-8 assay according to manufacture protocol. Data is normalized to pancreatic cancer cells (Panc-1) treated with non-conditioned media. All experiments were repeated 3-4 times and representative data is presented. Data represents mean + SEM. Statistics generated by t-test, *p < 0.05, **p < 0.01.

Subsequently, to evaluate the effects of miR-29 overexpression in PSCs on cancer growth, we transfected PSCs with control or miR-29 mimics and co-cultured them with pancreatic cancer cells. Direct co-culture of PSCs overexpressing miR-29a or b with pancreatic cancer cells caused a significant decrease in the ability of the cancer cells to form colonies (Fig. 6a and Supp Fig. 14a) and reduced stromal deposition associated with cancer colonies (Supp Fig. 14b). To elucidate the underlying mechanism that leads to a decrease in pancreatic cancer colony formation, we evaluated the autocrine and paracrine effects of miR-29 on the viability of PSCs and pancreatic cancer cells, respectively. For autocrine effects of miR-29, we transfected PSCs with control or miR-29 mimics, and cell viability was monitored for up to 96 hours post-transfection. Overexpression of miR-29 did not reduce PSC viability compared to cells transfected with control mimics (Supp Fig. 15). To determine the paracrine effects of miR-29 on cancer cell viability, pancreatic cancer cells were cultured in conditioned media collected from PSCs transfected with control or miR-29 mimics. We observed a significant decrease in viability of pancreatic cancer cells growing in conditioned media collected from miR-29 transfected PSCs compared to PSCs transfected with control mimics (Fig. 6b and Supp Fig. 16). Finally, to evaluate the effect of miR-29 overexpression in PSCs on anchorage independent growth of pancreatic cancer cells, we co-cultured miR-29 transfected PSCs with cancer cells in soft agar assays. We observed a decrease in anchorage independent growth of pancreatic cancer cells when co-seeded with miR-29a overexpressing PSCs compared to PSCs transfected with control mimics (Supp Fig. 17). Overall, overexpression of miR-29 in PSCs caused a decrease in stromal/ECM protein accumulation and cancer colony growth. However, the long-term consequences of miR-29 overexpression in stromal deposition and cancer progression need to be further evaluated *in vivo.*

### TGF-β1-mediated downregulation of miR-29 in PSCs is SMAD3 dependent

**Figure 7:**
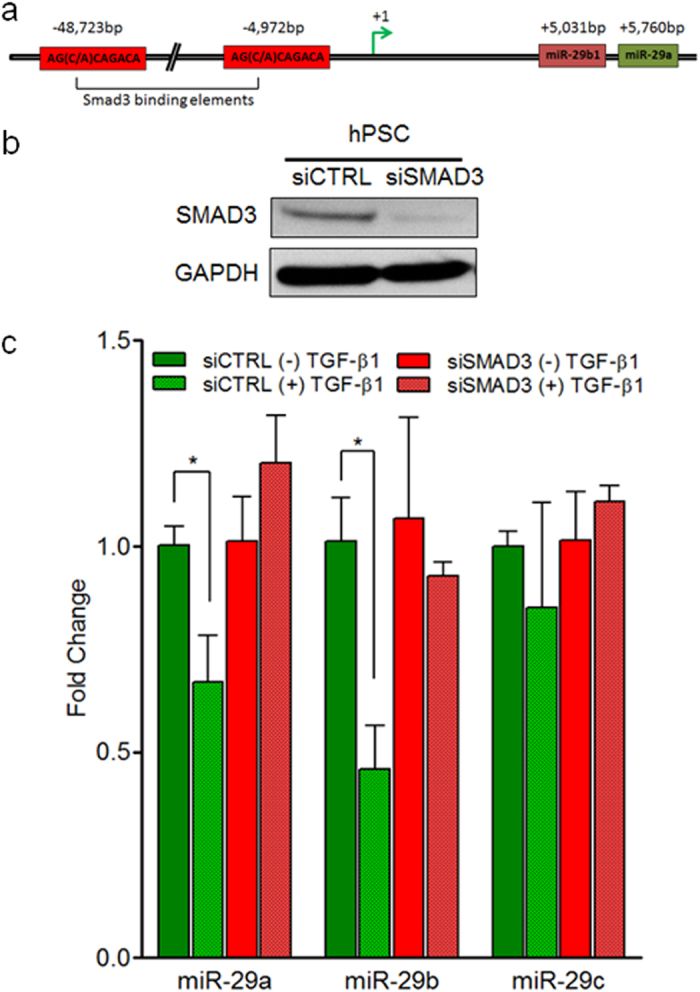
TGF-β1-mediated downregulation of miR-29 expression in PSCs is SMAD3 dependent. (**a**) Predicted SMAD3 binding sites upstream of miR-29a/b1 promoter: there are two SMAD3 binding elements (S3BE) 5 kb and 49 kb upstream of the transcription start site of the miR-29a/b1 loci on chromosome 7. SMAD3 binds specifically to CAGA boxes: AG(C/A)CAGACA and regulates neighboring gene expression. (**b**) siSMAD3 efficiently reduces endogenous SMAD3 protein levels in hPSCs. hPSCs growing in culture were transfected with 50 nM non-targeting siRNA (siCTRL) or siSMAD3. 24 hours post-transfection, total proteins were harvested and subjected to western blot analysis to determine SMAD3 expression levels. GAPDH was used as a loading control. (**c**) SMAD3 knockdown abrogates TGF-β1-mediated miR-29 repression. qPCR analysis of miR-29 levels in TGF-β1-activated hPSCs transfected with siCTRL and siSMAD3. hPSCs were transfected with 50 nM siCTRL or siSMAD3. 24 hours post-transfection, cells were challenged with 10 ng/ml TGF-β1 for 24 hours and subjected to qPCR for miR-29 expression levels. All experiments were repeated three times and representative data is presented. Data are presented as mean + SEM; n = 3, statistics generated by t-test, *p < 0.05.

We next sought to identify the underlying mechanism of TGF-β1 activated loss of miR-29 in PSCs. Others have previously identified SMAD3 binding elements near the miR-29a/b-1 locus on chromosome 7 upstream of the miR-29a/b1 transcription start site^42^ (Fig. 7a). We subsequently knocked down SMAD3, a major downstream effector molecule of TGF-β1, which is known to directly regulate miR-29 expression in myoblast and renal cells^43^,^44^ and confirmed the loss of SMAD3 protein in transfected PSCs by western blot analysis (Fig. 7b). PSCs transfected with control siRNA displayed a significant downregulation of miR-29a and miR-29b upon TGF-β1 stimulation (Fig. 7c). Whereas in the absence of SMAD3, PSCs no longer showed a significant change in miR-29 (Fig. 7c), suggesting that the ability of TGF-β1 to suppress miR-29 expression is SMAD3 dependent. Our results, in conjunction with previous findings in muscle and kidney^25^,^45^ suggests that TGF-β1-mediated loss of miR-29 in PSCs may be due to the direct binding of SMAD3 to miR-29 promoter elements.

## Discussion

Dense, fibrotic stroma is a histopathological hallmark of pancreatic cancer, and it precludes drug delivery to the tumor bed^3^,^4^. Activated PSCs are major contributors of fibrotic stromal reaction and are known to interact with cancer cells and promote PDAC tumor progression^6^,^7^. Quiescent PSCs normally located within the periacinar/ductal regions of the pancreas are activated in response to autocrine and paracrine pro-inflammatory cytokines/growth factors and secrete excessive amount of ECM proteins, a major component of PDAC stroma^16^. TGF-β1 is a pro-tumorigenic/fibrotic growth factor secreted from cancer cells and injured acinar cells and is known to be a vital contributor of PSCs activation^16^,^18^. The upregulation of TGF-β1 in PDAC has been well documented and perpetuates the stromal reaction^13^,^14^.

Although previous studies have shown that miR-29 is reduced in TGF-β1 signaling and promotes fibrosis of various organs^43^,^44^, the role of miR-29 in the context of PSCs and PDAC fibrotic stroma has yet to be elucidated. In our study, we show for the first time that TGF-β1 acts as a negative regulator of miR-29 in PSCs, while simultaneously upregulating ECM proteins: collagens, laminin, and fibronectin. Through qPCR analysis, we observed a predominant downregulation of miR-29a and miR-29b family members in both mouse and human PSCs upon TGF-β1-mediated activation. In muscle and kidney fibrosis, TGF-β1 is known to downregulate miR-29 expression in myoblasts^43^ and renal cells^44^ through SMAD3 binding elements located upstream of miR-29 promoter region and promote fibrosis. Similarly, we found that TGF-β1-mediated miR-29 repression in PSCs is SMAD3 dependent. In addition to PSCs, TGF-β1 activated pancreatic fibroblasts^5^ (additional stromal cells responsible for stromal reaction), also displayed a loss of miR-29. Interestingly, we found the downregulation of all three miR-29 family members in TGF-β1 stimulated CAFs isolated from PDAC patient tumors. miR-29 family members encoding genes reside on two different human chromosomes with miR-29a/b1 loci on Chromosome 7 and the miR-29c/b2 loci on Chromosome 1 (Fig. 5a)^42^, lending to the possibility that TGF-β1 may elicit downstream effects to inhibit both miR-29 encoding loci in CAFs but not in PSCs and normal pancreatic fibroblasts. Our findings implicate a differential effect of TGF-β1 in regulation of miR-29 expression in CAFs compared to PSCs/pancreatic fibroblasts that warrants further studies and may reveal differential regulatory mechanisms between miR-29 family members.

Subsequent investigation of miR-29 expression patterns in the pancreata of spontaneous tumor forming PDAC mouse model carrying initiating oncogene Kras^G12D^ (KC)^39^ and in PDAC patient tumor biopsies revealed similar findings of miR-29 loss of expression. We found a global decrease in expression of all three miR-29 family members in Kras^G12D^ murine pancreata and human PDAC in conjunction with an increase in pancreatic fibrosis and stromal deposition associated with PDAC tumors. Although we observed a global loss of miR-29, these findings did not implicate whether the loss of miR-29 is specific to PSCs and epithelial cells associated with murine and human PDAC. Thus, we performed *in situ* hybridization to assess PSC and epithelial cell specific miR-29a expression by co-staining KC mice pancreata and human PDAC tumors with GFAP, a marker specific to PSCs/fibroblasts^40^ and CK19, a marker specific to epithelial cells^41^. Accordingly, we found a similar loss of miR-29a in PSCs of KC mice and PDAC patient samples. These findings further confirm that the loss of miR-29 is consistent in PSCs associated with PDAC stromal reaction. Nevertheless, our results do not document the underlying mechanisms for the loss of miR-29 expression in K-Ras^G12D^ activated murine pancreata and human PDAC tumors, but our findings advocate for future functional studies *in vivo* to examine miR-29 expression patterns in disease onset and progression in various etiologies associated with human PDAC.

Stromal ECM proteins such as collagens and laminin are direct miR-29 targets. These proteins in addition to fibronectin, another stromal abundant protein, interact with cancer cells and enhance tumor progression of various malignancies^46^,^47^ including pancreatic cancer^48^. Although miR-29 has been shown to suppress fibrosis of several different organs, including heart^34^, liver^24^, lung^26^, kidney^25^, and muscle^30^ by negatively regulating ECM protein expression, the function of miR-29 in PSC-mediated ECM protein expression has yet to be validated. Using miR-29 gain and loss-of-function studies, we found that miR-29 suppresses its direct ECM targets collagen1a1, collagen 3a1, and laminin in activated PSCs. Surprisingly, we also found that miR-29 inhibits fibronectin expression, even though fibronectin does not contain canonical miR-29 binding sites within its 3′-untranslated region (Targetscan, miRanda, and PicTar). As a result, we sought to find an indirect mechanism for miR-29-mediated fibronectin inhibition in TGF-β1 activated PSCs. Interestingly, we found that ectopic expression of miR-29 in TGF-β1 activated PSCs reduced SMAD2 phosphorylation, implicating that miR-29 may act as an auto-inhibitory feedback regulator within TGF-β1 signaling. TGF-β1 is known to play a key role in PDAC pathogenesis, and its upregulation has been well documented in PDAC^14^ and pancreatitis^15^, a major risk factor of PDAC. As a consequence, TGF-β1 has emerged as an appealing therapeutic target, and numerous therapeutic strategies have been developed to inhibit TGF-β1 signaling via ligand inhibition and ligand/receptor interactions^49^. Our findings reveal miR-29 to be a potential downstream inhibitor of TGF-β1 signaling.

In addition to the fibrotic stromal reaction, PSCs are known to interact with pancreatic cancer cells and promote tumor progression and metastasis. In our functional studies, restored expression of miR-29 in PSCs caused a decrease in cancer colony growth and stromal protein accumulation associated with co-cultures. Our data suggests that miR-29 overexpression reduced cancer colony growth by inhibiting the paracrine effects of PSCs on cancer cells, as we observed a decrease in pancreatic cancer cell viability. Admittedly, the precise mechanism associated with the paracrine effect of miR-29 overexpression in PSCs on cancer cell growth has yet to be elucidated, and future studies would further shed light on the role of miR-29 in tumor-stromal interactions. A large body of experimental evidence documents the role of extracellular matrix proteins such as collagen and laminin in cancer cell proliferation and drug resistance^50^,^51^,^52^. Although we observed that the overexpression of miR-29 in PSCs reduced stromal accumulation associated with cancer colonies, additional studies are required to elucidate the direct mechanistic role of miR-29 in ECM-mediated pancreatic cancer progression.

As fibrotic stroma impairs the efficacy of chemotherapeutics and promotes PDAC progression, it is considered as an attractive therapeutic target in developing effective treatment strategies to target PDAC. Current, anti-stromal therapies to date have sought to deplete the reactive stroma. However, these approaches failed to improve patient survival and are often associated with toxicity. Furthermore, recent studies show that stroma impedes tumor growth, and its complete inhibition accelerated disease progression^10^,^11^. However, inactivating PSCs by modulation of a key transcriptional regulator suppressed the reactive stroma, increased tumor response to chemotherapy and survival^12^. This evidence suggests that normalizing reactive stroma is a safe and efficacious treatment strategy, as opposed to completely ablating the reactive stroma.

In our studies using *in vitro* and *in vivo* models and PDAC patient biopsies, we observed a loss of miR-29 in PSCs and fibroblasts, the critical stromal cells responsible for fibrotic stroma. Restored expression of miR-29 in PSCs suppressed major stromal protein expression (collagens, laminin and fibronectin) and inhibited cancer cell growth in co-culture. While the long-term consequences of miR-29 overexpression or miR-29 loss-of-function on PDAC progression/metastasis and its role in cancer cells and the tumor microenvironment remain to be investigated *in vivo*, our findings indicate that the efficacy of stroma-targeted therapy in PDAC may also be dictated by stromal miRNA expression and function. We anticipate that the restored expression of miR-29 in stromal cells reduces the stromal protein accumulation and cancer growth and enhances the drug delivery to the inner tumor core. Furthermore, increasing evidence indicate miR-29 plays a vital role in cancer pathogenesis^32^, tumor microenvironment, and metastasis^33^. In contrast to pharmacological approaches, the use of miR-29 as a therapeutic agent may be more effective in targeting reactive stroma, as a single miRNA regulates the expression of several genes associated with disease mechanisms^23^. Thus, restored expression of a critical miRNA is therapeutically beneficial and targets multiple cellular pathways associated with disease processes. In our previous work, we demonstrated that the replacement of a single missing miRNA suppressed tumor progression with no toxicity and off-target effects^53^.

Thus far, no studies have interrogated the relevance of miR-29 in the context of PDAC stroma. Based on our *in vitro*, *in vivo*, and clinical observations in stromal cells and functional studies, our results indicate that miR-29 plays a critical role in stromal deposition and inhibits the pro-growth effects of PSCs on pancreatic cancer colony formation. Our findings raise the possibility that miR-29 could serve as an anti-stromal therapeutic agent in the context of PDAC. A large body of evidence demonstrates the pleiotropic role of miRNAs in fibrotic process, cancer pathogenesis and metastasis, and their potential use as therapeutic agents for cancer^53^,^54^,^55^, fibrosis^56^, and other human diseases^57^. Some miRNA-based drugs have already reached clinical trials^57^,^58^ or are in advanced stage of pre-clinical development^59^,^60^, indicating the feasibility of miR-29 in prospective clinical applications. This new and substantially different approach is expected to overcome the problems associated with other means of modulating the stroma and result in an effective approach to improve drug delivery to the tumor bed. Future work aimed at determining the role of miR-29 in pancreatic cancer cells, patient survival, and its biological functions *in vivo* will allow us to further understand the role of miR-29 in PDAC development and progression and ultimately determine its prognostic and therapeutic applicability to target PDAC.

## Methods

### Mice

*Kras*^*G12D*^; *Pdx1-Cre* (KC) mice were generated as described^39^. Conditional *LSL-Kras*^*G12D*^ mice were crossed with *Pdx1-Cre* animals to generate the KC mice. All animal housing, use, and surgical procedures were carried out in accordance with the regulatory guidelines set by Guide for the Care and Use of Laboratory Animals of the National Institutes of Health. All animal protocols were reviewed and approved by the Indiana University (IU) Animal Care and Use Committee.

### Cell lines

Immortalized mouse pancreatic stellate (mPSC) cell lines^61^ were a kind gift from Dr. Raul Urrutia at Mayo Clinic, Rochester, Minnesota. Primary human PSCs were obtained from ScienCell Research Laboratories (Carlsbad, California), grown routinely in Dulbecco’s Modified Eagle Medium (DMEM) (Life Technologies, 11965-092) supplemented with 10% fetal bovine serum (FBS), 100 units ml^−1^ penicillin, and 100 mg ml^−1^ streptomycin. Primary human fibroblasts were purchased from Vitra Biopharma (Golden, CO), and were routinely cultured in their recommended media (VitroPlusII). Primary cancer associated fibroblasts (CAFs) isolated from PDAC patients were confirmed by α-SMA staining and authentication was performed by IDEXX-Radil (Columbia, MO). CAFs were maintained in DMEM/F-12, GlutaMAX Supplement (Life Technologies, 10565-018) plus 10% FBS, 100 units ml^−1^ penicillin, and 100 mg ml^−1^ streptomycin. All primary cell lines were studied prior to reaching seven passages. Human pancreatic carcinoma cell lines Panc-1 and MIA PaCa-2 were kind gifts from Karen E. Pollok of Indiana University School of Medicine and IU Simon Cancer Center. Both were grown routinely in DMEM supplemented with 10% FBS, 100 units ml^−1^ penicillin, and 100 mg ml^−1^ streptomycin.

### Patient Tissue Procurement

This study was reviewed and approved by the Indiana University-Purdue University Indianapolis (IUPUI) Institutional Review Board (IRB) at Indianapolis, IN, USA (IUPUI IRB# 1303011057). Tissue biopsies and associated clinical data were obtained from IU Simon Cancer Tissue Bank and IU Department of Pathology. Both IU Simon Cancer Tissue Bank and IU Department of Pathology procured tissue samples and informed consent was obtained from human subjects in compliance with IUPUI IRB policies. IUPUI IRB is accredited by the Association for the Accreditation of Human Research Protection Programs (AAHRPP). Quality and integrity of paraffin embedded or fresh pancreatic tissue biopsies were analyzed by a certified pathologist with hematoxylin and eosin staining. Control samples that were biopsied from patients with conditions unrelated to pancreatic disease or from an unaffected area of the pancreas from patients with pancreatitis were confirmed via histopathological analysis. Corresponding patient clinical data of each sample is provided in the Supplementary Information (Supp Table 1 and Table 2).

### RNA Extractions

Total RNA was extracted from formalin fixed paraffin embedded (FFPE) tissue samples (two 10 μm thick sections) using the MagMax^TM^ FFPE total nucleic acid isolation kit (Ambion, Life Technologies, 4463365) or from frozen tissues using Rino Bullet Blender beads (MidSci, CAP329) followed by Trizol (Life Technologies, 15596018) extraction. RNA was extracted from cells *in vitro* by Trizol extraction according to the manufacturer’s protocols. The quantity and purity of RNA was determined by OD260/280 reading using a Nanodrop spectrophotometer.

### qPCR analysis

Mature miR-29 family member expression levels were measured by TaqMan MicroRNA Assays (Applied Biosystems) for miR-29a (ID:002112); miR-29b (ID:000413); and miR-29c (ID:000587). U6 snRNA (ID:001973) was used as a reference gene to normalize the relative amount of miRNA, and samples were analyzed using ABI 7500 real time PCR machine. Alpha smooth muscle actin (α-SMA), connective tissue growth factor (CTGF), collagen 1a1 (COL1A1), laminin (LAMC1), and fibronectin (FN1) levels were measured using gene specific qPCR primer probes purchased from Solaris Probes. RNA samples were reverse transcribed using random primers, and α-SMA (AX-061937-00-0100); CTGF (AX-040018-00-0100); COL1A1 (AX-042068-00-0100); LAMC1 (AX-043874-00-0100); and FN1 (AX-043446-00-0100) expression levels were measured using cyclophilin B (PPIB) (AX-048843-00-0100) as an internal control. Samples were run in triplicates with 0.2 thresholds, and the ΔΔCT method was used for relative expression analysis. For miR-29 expression analysis in PDAC patient tumors, total RNA was isolated from FFPE tissue sections (two 10 μm) of normal controls (n = 10) or PDAC patient tumors (n = 15) with 35–80% stroma and miR-29 family expression was analyzed by qPCR as described above.

### Immunoblotting

mPSCs and hPSCs were lysed in lysis buffer (50 mM HEPES, 150 mM NaCl, 10% glycerol, 1% Triton-X, 1.5 mM MgCl_2_, 1 mM EGTA, 100 mM NaF, 10 mM NaPPi, Na O Vanidate, ZnCl_2_, PMSF, protease inhibitor) and protein samples were ran through SDS-PAGE. Proteins were transferred to polyvinylidene fluoride membrane (PVDF), blocked in 10% dried non-fat milk, and subsequently probed with antibodies against collagen 1alpha1 (Santa Cruz, sc-8784-R, 1:500), collagen 3alpha1 (Santa Cruz, sc-8781, 1:500), laminin gamma-1 (Santa Cruz, sc-5584, 1:5000), fibronectin (Santa Cruz, sc-9068, 1:500), α-tubulin (Calbiochem, CP06, 1:3000), GAPDH (Millipore, MAB374, 1:10,000), and β-actin (Santa Cruz, sc-47778, 1:2000), using corresponding HRP conjugated goat anti-rabbit (Santa Cruz, sc-2004), goat anti-mouse (Santa Cruz, sc-2005), or donkey anti-goat (Santa Cruz, sc-2020) secondary antibodies. Proteins were visualized and quantified using chemiluminescent detection (GE Healthcare, Amersham ECL) and exposure to x-ray film (Thermo Scientific, CL-X Posure Film). The intensity for each band was densitometrically quantified and normalized against loading control using ImageJ software.

### Transfection

Exponentially growing mPSCs or hPSCs were seeded in 6 well plates at 1 × 10^5^ cells per well, serum starved for 24 hours, and treated with 10 ng/ml of TGF-β1 for 24 hours before or after transfection with 20 nM mimics using DharmaFECT®1 (Life Technologies, T-2001-01) per their protocol. For immunoblotting, cells were either transfected with 20 nM mimic control (Life Technologies, CN-001000-01), mimic-29a (Life Technologies, C-300520-05), or mimic-29b (Life Technologies, C-300520-05). For LNA transfection, 1 × 10^5^ serum starved and TGF-β1 treated hPSCs were transfected with 50 nM of LNA-29 (Exiqon, 450039) or LNA-control (Exiqon, 199006) using DharmaFECT^®^1. 24 hours post-transfection, protein was harvested and subjected to western blot analysis. For studies involving siSMAD3, 1 × 10^5^ hPSCs/well (6-well plate) growing in culture were transfected with 50 nM non-targeting siRNA control (Life Technologies, D-001810-01-05) or siSMAD3 (Life Technologies, L-020067-00-005) using DharmaFECT^®^1 to inhibit endogenous SMAD3 levels in PSCs. 24 hours post-transfection, total proteins were harvested and subjected to western blot analysis to verify SMAD3 knockdown efficiency. For miR-29 expression analysis in TGF-β1-activated hPSCs transfected with siCTRL and siSMAD3, hPSCs were transfected with 50 nM siCTRL or siSMAD3 as described. 24 hours post-transfection, cells were serum starved for 24 hours, challenged with 10 ng/ml of TGF-β1 for 24 hours, and subjected to Trizol RNA isolation. miR-29 expression levels were analyzed via qPCR as described above.

### Co-culture

1 × 10^5^ mPSCs were transfected with either 20 nM mimic control, 20 nM mimic-29a, or 20 nM mimic-29b and plated simultaneously with 100 pancreatic cancer cells (MIA PaCa2 or Panc-1) in a 6 well plate. Co-cultures were allowed to grow for 10 days, and were fixed and stained with crystal violet solution (0.05% w/v crystal violet, 1% formaldehyde, 1x PBS, 1% MeOH) or Sirius Red. Cancer colonies greater than 50 cells were counted under phase contrast microscopy.

### Collection of PSC Conditioned Media

To obtain miR-29 and control mimic transfected PSC conditioned media, exponentially growing mPSCs were seeded in a 6 well plates at 1 × 10^5^ cells per well, serum starved for 24 hours, and treated with 10 ng/ml of TGF-β1 for 24 hours before transfection with 20 nM control or miR-29 mimics using DharmaFECT®1 per their protocol. 24 hours post-transfection, cells were washed twice with PBS and incubated with DMEM with 0.2% BSA for 48 hours at 37 °C. Conditioned media was collected from PSCs and centrifuged at 1500 rpm for 10 min at 4 °C. Supernatant was collected and then filtered using a 0.22 μm sterile filter (Fisher Scientific, 09-719A). In parallel, non-conditioned media control was collected from a 6 well plates with no PSCs.

### Cell Viability

1,000 PSCs were plated per well in a 96 well plate. Cells were serum starved for 24 hours, and treated with 10 ng/ml of TGF-β1 for 24 hours before transfection with 20 nM control or miR-29 mimics using DharmaFECT®1 per their protocol. Cell viability was measured at 24, 48, 72, and 96 hours after plating the assays by adding 10 ul Cell Counting Kit-8 (Dojindo #CK04) reagent and absorbance was measured at 450 nm. For cancer cell viability,

 5,000 pancreatic cancer cells (MIA PaCa-2 or Panc-1) were plated per well in a 96-well plate. Cancer cells were washed with PBS, and treated with PSC conditioned media for 24 and 48 hours. Cell viability was measured post-treatment by adding 10 ul of Cell Counting Kit-8 reagent and absorbance was measured at 450 nm.

### Soft agar assays

24 hours post-transfection with 20 nM control or miR-29 mimics, 50,000 mPSCs were co-seeded with 5,000 pancreatic cancer cells (MIA PaCa-2 or Panc-1) per well in a 6 well plate containing 0.5% top agarose and 1% bottom agarose (BioRad, #162-0137). After 7 days, colonies were stained with crystal violet and were counted under low power for positive colonies (>6 cells/colony).

### Histology

FFPE pancreata of KC and C57BL/6 mice or normal control and PDAC patients were sectioned (5 μm) and stained with H&E for gross histological analysis. Tissue sections were also stained with Sirius Red to estimate collagen and the degree of fibrosis. ImageJ analysis was used to quantify Sirius Red positive collagen area in the pancreatic sections (four 20X random images/animal).

### Immunofluorescence

1 × 10^5^ mouse PSCs, human PSCs or primary human fibroblasts were plated on collagen coated cover slips in 6-well plates and grown to ~80% confluency. Cells were washed with PBS, fixed in 4% paraformaldehyde at 37 °C and then permeabilized with 0.25% Tween-20 in PBS (PBST). Subsequently, cells were blocked with 1% BSA in PBS-T at room temperature and α-SMA primary antibody (α-SMA, Novus Biologicals, NB600-531, 1:500) and secondary antibody (Goat-α-Rabbit-594, Molecular Probes, 11012, 1:500) were used to detect intracellular α-SMA levels. Slides were coversliped using VectaShield mounting medium with DAPI (H-1200).

### *In Situ* Hybridization

FFPE pancreata of C57BL/6 control mice at 2 and 10 months (n = 3/group) and KC mice at 1, 4, and 9 months (n = 3/group) were sectioned and subjected to *in situ* hybridization as previously described^62^. FFPE pancreatic tissue sections (5 μm) from normal control and PDAC patients (n = 4/group) were also subjected to miR-29 *in situ* hybridization as described. Slides were hybridized with 50 nM 5′-biotin labeled U6 snRNA LNA probe (Exiqon, 99002-03) and 5′ and 3′ DIG labeled miR-29a LNA probe (Exiqon, 10000-899999-15) for 90 minutes at 55 °C. Subsequently, slides were probed with GFAP (Novus Biologic, NB300-141, 1:200) or CK19 antibody (Abcam, ab52625, 1:200) and stained with secondary antibodies conjugated to HRP to facilitate Tyramide signal amplification (TSA) reaction: goat anti-rabbit IgG (Santa Cruz, sc-2004, 1:500–1:1000), anti-Dioxigenin (Abcam, ab6212, 1:200), and Streptavadin (Thermo Scientific, #21130, 1:5000). Slides were then stained with Hoechst NucRed^TM^ Dead 647 (Life Technologies, #R37113) to stain nuclei. 20X images were taken using DeltaVision Core confocal microscope, projected in four channels, pseudo-colored, and merged. Corrected total cell fluorescence (CTCF) of miR-29a (green) was quantified in six or more randomly selected GFAP-positive PSCs/fibroblasts or CK19-positive epithelial cells using ImageJ analysis as previously described^63^.

### Statistical analysis

Student’s t-test was used for statistical analysis. Data is presented as mean and error bars are represented as standard error of the mean.

## Additional Information

**How to cite this article**: Kwon, J. J. *et al.* Pathophysiological role of microRNA-29 in pancreatic cancer stroma. *Sci. Rep.* **5**, 11450; doi: 10.1038/srep11450 (2015).

## Supplementary Material

Supplementary Information
