# Supplementary material for: Pathophysiological role of microRNA-29 in pancreatic cancer stroma
Source: Sci Rep. 2015 Jun 22;5:11450. doi: 10.1038/srep11450 (PMC4476113; doi:10.1038/srep11450)
Supplement: Supplementary Information [file srep11450-s1.pdf]

Supplementary Information

Pathophysiological role of microRNA-29 in pancreatic cancer stroma

Authors: Jason J Kwon[1#], Sarah C Nabinger[1#], Zachary Vega[2], Smiti S Sahu[1,3], Ravi K Alluri[1], Zahi Abdul Sater[4], Zhangsheng Yu[5], A Jesse Gore [6,7,8], Grzegorz Nalepa[1,4,9,10], Romil Saxena[3], Murray Korc[4,6,7,8], and Janaiah Kota[1,7,8*]

[1]Department of Medical and Molecular Genetics, Indiana University School of Medicine (IUSM), Indianapolis, IN, USA

[2]Wabash College, Crawfordsville, IN, USA

[3]Department of Pathology, IUSM, Indianapolis, IN, USA

[4]Department of Biochemistry and Molecular Biology, IUSM, Indianapolis, IN, USA

[5]Department of Biostatistics, IUSM, Indianapolis, IN, USA

[6]Department of Medicine, IUSM, Indianapolis, IN, USA

[7]The Melvin and Bren Simon Cancer Center, IUSM, Indianapolis, IN, USA

[8]Center for Pancreatic Cancer Research, Indiana University and Purdue University-Indianapolis (IUPUI), Indianapolis, IN, USA

[9]Department of Pediatrics, Herman B Wells Center for Pediatric Research, IUSM, Indianapolis, IN, USA

[10]Division of Pediatric Hematology-Oncology, Bone Marrow Failure Program, IUSM, Indianapolis, IN, USA

#These authors contributed equally to this work

[*]**Correspondence to**: jkota@iu.edu

Journal: Scientific Reports

Article Title:	**Pathophysiological role of microRNA-29 in pancreatic cancer stroma**
Corresponding Author:	Janaiah Kota

**Supplementary Item & Number**	**Title or Caption**
Supplementary Figure 1	Model of pancreatic ductal adenocarcinoma (PDAC) and tumor-stromal interactions
Supplementary Figure 2	miR-29 expression analysis in an additional mouse PSC cell line
Supplementary Figure 3	TGF-β1 mediated activation of human PSCs leads to an increase in pSMAD2/3 expression levels
Supplementary Figure 4	TGF-β1 activated PSCs exhibit increased expression of ECM components at the mRNA and protein level
Supplementary Figure 5	Pancreatic tissues from PDAC patient samples display a high degree of fibrosis
Supplementary Figure 6	miR-29a is the most abundantly expressed miR-29 family member in pancreatic stellate cells, pancreatic ductal epithelial cells, and the whole pancreas.
Supplementary Figure 7	miR-29a is decreased in GFAP-positive PSCs in KC mice
Supplementary Figure 8	miR-29a is decreased in CK19-positive epithelial cells in KC
Supplementary Figure 9	Mouse and human PSCs transfected with miR-29 mimics, have increased miR-29a and miR-29b expression
Supplementary Figure 10	Ectopic expression of miR-29 mimics reduce ECM components in TGF-β1 activated mouse PSCs
Supplementary Figure 11	miR-29 suppresses SMAD2 activation in mouse PSCs
Supplementary Figure 12	miR-29-LNA efficiently knockdown all endogenous miR-29 family members (miR-29a, miR-29b, and miR-29c) in mouse and human PSCs
Supplementary Figure 13	miR-29 knockdown increases direct miR-29 ECM target proteins in mouse PSCs
Supplementary Figure 14	Ectopic expression of miR-29 in PSCs causes reduced cancer colony formation in direct co-cultures
Supplementary Figure 15	Ectopic miR-29 expression does not affect PSC viability.
Supplementary Figure 16	Conditioned media of PSCs expressing miR-29 decreases pancreatic cell viability
Supplementary Figure 17	miR-29a decreases the effect of PSCs on anchorage independent growth of pancreatic cancer cells
Supplementary Table 1	Summary of clinical characteristics of the human subjects
Supplementary Table 2	Detailed demographic and clinical characteristics of the human subjects

Supplementary Figure 1

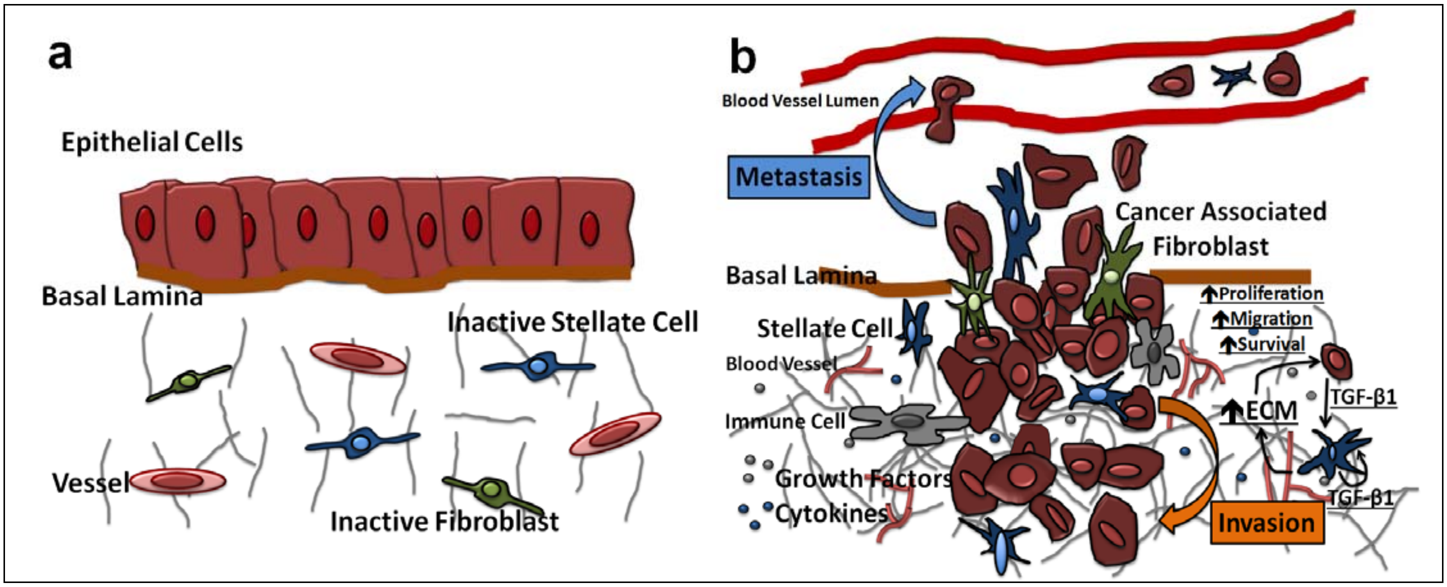

Supp Figure 1: Model of pancreatic ductal adenocarcinoma (PDAC) and tumor-stromal interactions.

(**a**) Depiction of healthy pancreatic epithelial cells (red), fibroblasts (green), pancreatic stellate cells (blue), and blood vessels (red).

(**b**) During carcinogenesis, both pancreatic cancer cells (dark brown) and pancreatic stellate cells (blue) release TGF-β1 and other pro-inflammatory cytokines/growth factors that activate pancreatic stellate cells to produce ECM proteins and increase fibrotic stromal deposition. A close interaction between stellate, cancer cells, and pro-inflammatory growth factors/cytokines contribute to the PDAC progression and metastasis.

Supplementary Figure 2

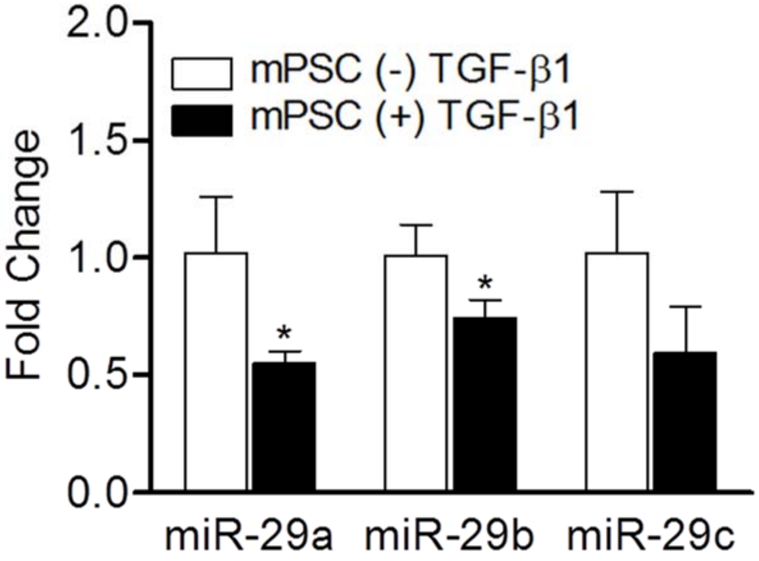

Supp Figure 2: miR-29 expression analysis in an additional mouse PSC cell line.
An additional immortalized mouse pancreatic stellate cell line (imPSC3) was serum starved, activated with 10ng/ml TGF-β1 for 24 hours, and subjected to RNA extraction. miR-29a, miR-29b, and miR-29c expression levels were analyzed by qPCR using U6 snRNA as an internal control. Data is presented as mean ± Standard Error of the Mean (SEM); n=3, statistics generated by t-test, *p<0.05.

Supplementary Figure 3

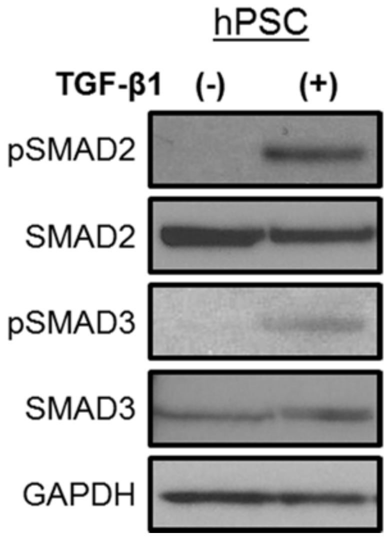

Supp Figure 3: TGF-β1 mediated activation of human PSCs leads to an increase in pSMAD2/3 expression levels.
hPSCs were serum starved for 24 hours and treated with 10ng/ml TGF-β1 for 1 hour. Total proteins were harvested and subjected to western blot analysis of phosphorylated SMAD2/3 (pSMAD2, pSMAD3) and total SMAD2/3. GAPDH was used as a loading control.

Supplementary Figure 4

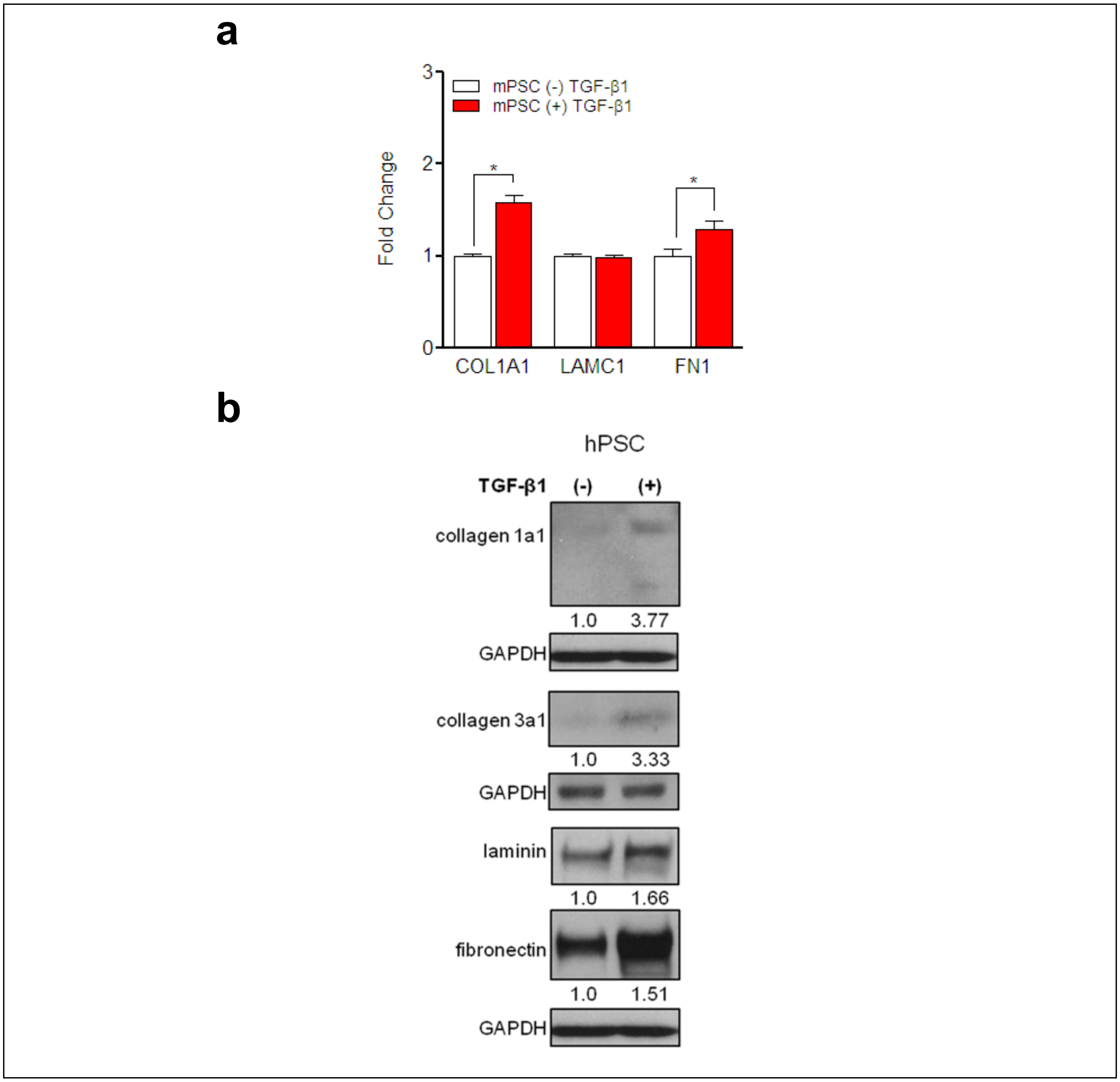

Supp Figure 4: TGF-β1 activated PSCs exhibit increased expression of ECM components at the mRNA and protein level.
(**a**) mPSCs were serum starved for 24 hours and then stimulated with 10ng/ml TGF-β1 for 24 hours. Total RNA was extracted and used to quantify miR-29 targets, collagen 1a1 (COL1A1), laminin (LAMC1), and fibronectin (FN1). Data is presented as mean + SEM; n=6/group, statistics generated by t-test, *p<0.05.
(**b**) Western blot analysis of ECM protein levels of collagen 1a1, collagen 3a1, laminin gamma-1 (laminin) and fibronectin in hPSCs which were serum starved and treated with TGF-β1 for 24 hours. GAPDH was used as loading control. Relative quantification of band intensities, normalized to loading controls, are shown below respective blotsKwon et al.

Supplementary Figure 5

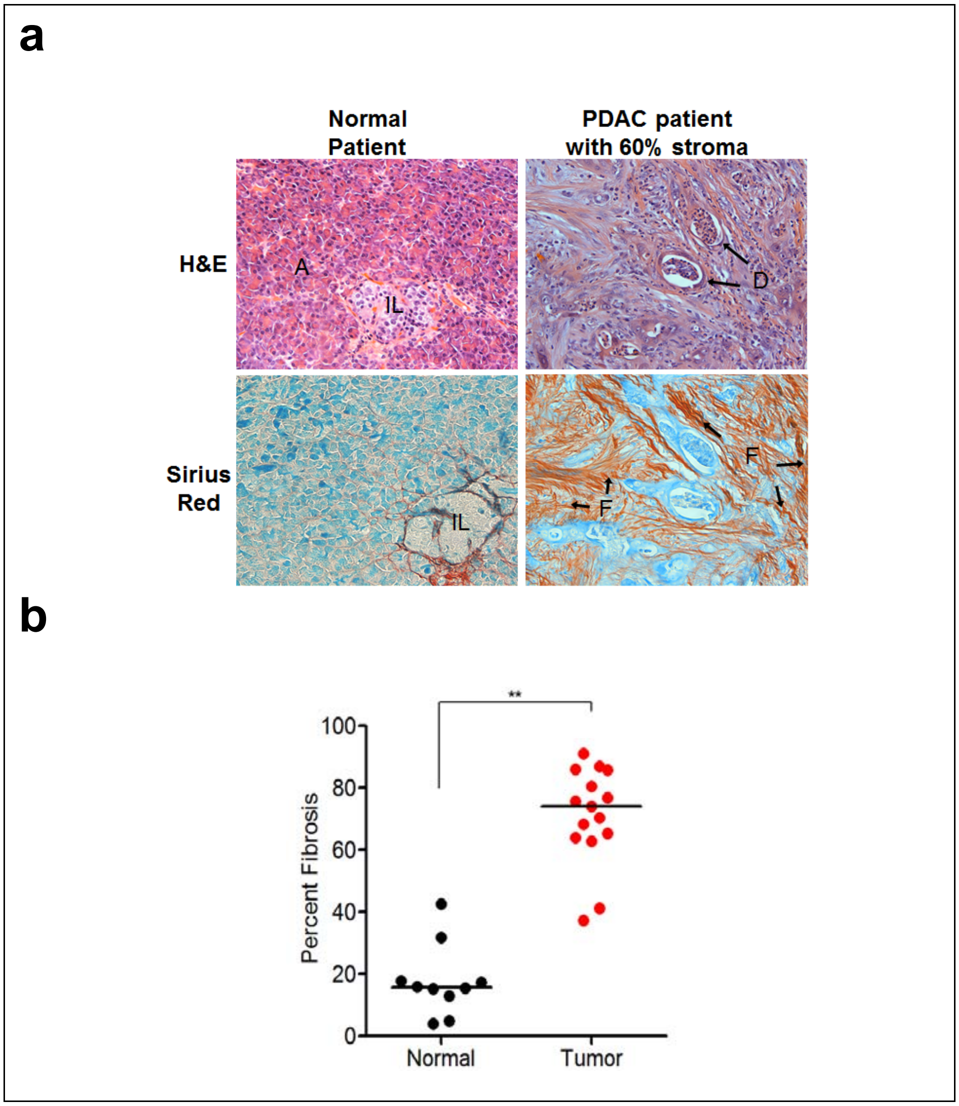

Supp Figure 5: Pancreatic tissues from PDAC patient samples display a high degree of fibrosis.

(**a**) Representative H&E and Sirius Red stained pancreatic sections from normal controls or PDAC patient tumors. Acinar cells (A), Islets of Langerhans (IL), pancreatic ducts (D), and prominent fibrosis (F) are demonstrated in the PDAC patients.

(**b**) Quantification of fibrosis/collagen in normal and PDAC patients. FFPE sections from normal and PDAC patient tumors were stained with H&E for gross histological analysis and Sirius Red to estimate the degree of fibrosis/collagen. Percent fibrosis in normal patient controls (n=10) and PDAC patient tumors (n=15) was calculated using Sirius Red positive pancreatic sections (four 20X random images/patient). ImageJ was used to quantify Sirius Red positive area. The mean percentage of fibrosis/collagen for each group is shown as horizontal lines. Statistics were generated using t-test, $^{**}p<0.01$.

Supplementary Figure 6

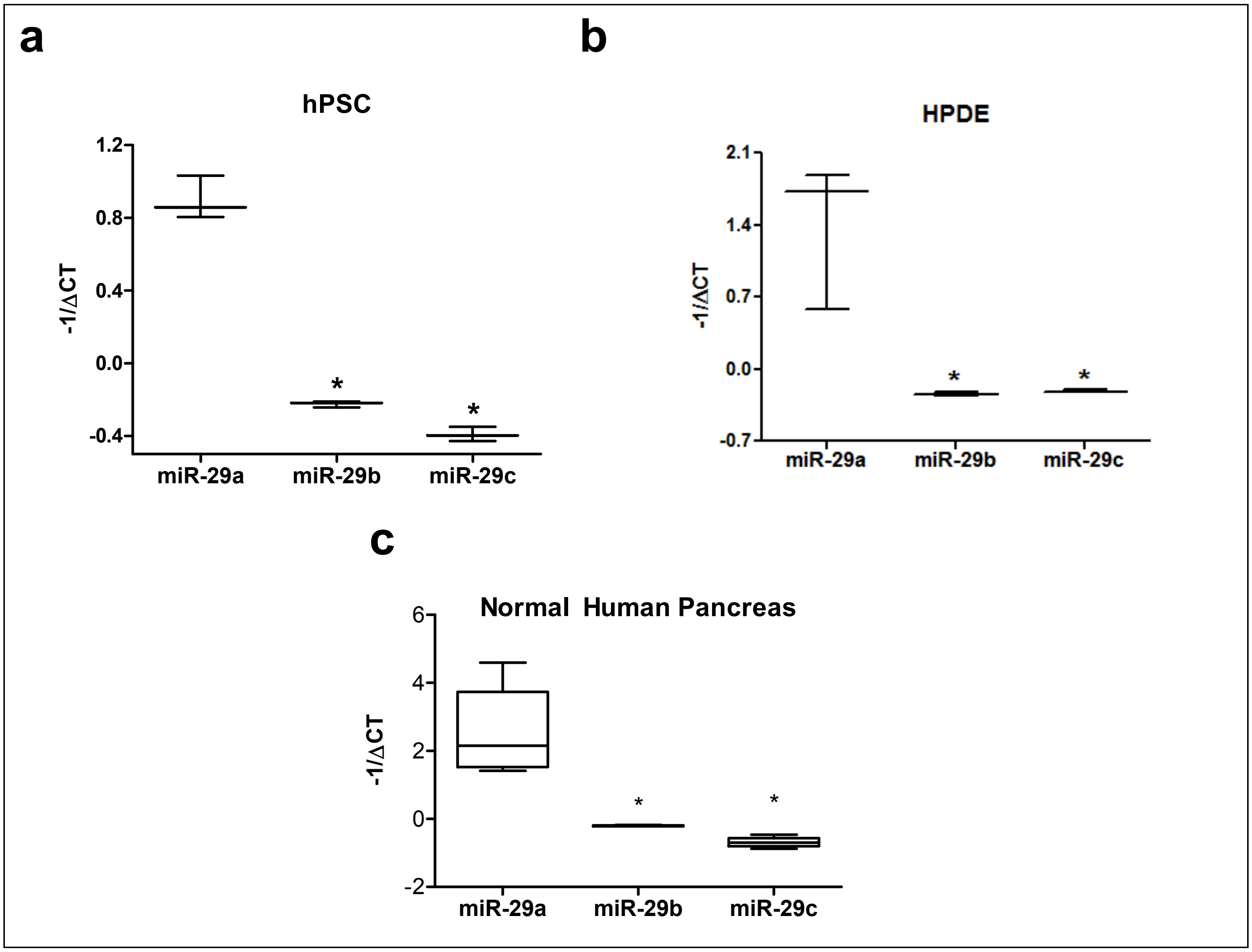

Supp Figure 6: miR-29a is the most abundantly expressed miR-29 family member in pancreatic stellate cells, pancreatic ductal epithelial cells, and the whole pancreas. miR-29a, -29b, -29c expression was measured in RNA isolated from (**a**) nascent hPSCs (serum starved 24hrs), (**b**) pancreatic ductal epithelial cells (HPDE) (n=3), or (**c**) normal human pancreas (n=5), by qPCR using U6 snRNA as an internal control. Delta CT (ΔCT) was calculated for each miR-29 family member to measure relative expression levels. Boxplots represent -1/ ΔCT of miR-29 expression levels in (hPSC, HPDE, or normal human pancreas,). Statistics calculated using student t-test, *$p<0.05$.

Supplementary Figure 7

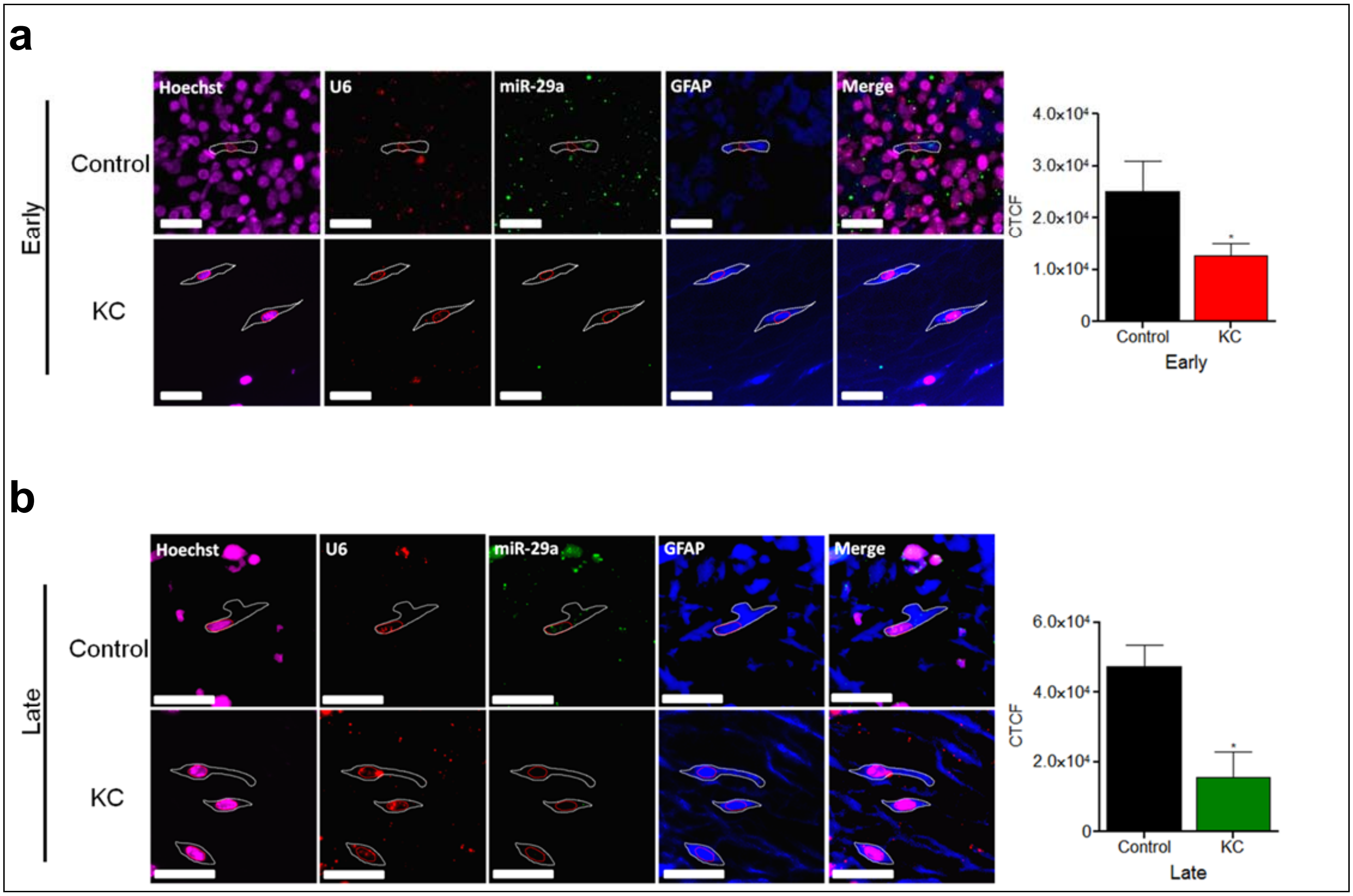

Supp Figure 7: miR-29a is decreased in GFAP-positive PSCs in KC mice.
In situ hybridization of miR-29a in pancreata of control mice (C57BL/6) and KC mice at early and late time points. FFPE pancreatic tissue sections collected at (**a**) early (2-4 months) and (**b**) late (9-10 months) time points from C57BL/6 control or KC mice (n=3/group/time point) were subjected to miR-29a *in situ* hybridization and images were generated using tyramide substrate amplification technique. Corrected total cell fluorescence (CTCF) of miR-29a was calculated for each animal by averaging six randomly selected GFAP-positive PSCs/fibroblasts using ImageJ analysis. Data represents the mean ± SEM; n=3; statistics generated by t-test, *p<0.05. Representative images are presented as a single channel, or merged (scale bar is 5μm, 20X magnification). Hoechst Nuclear stain (magenta), U6 (red), miR-29a (green), and GFAP (blue).

Supplementary Figure 8

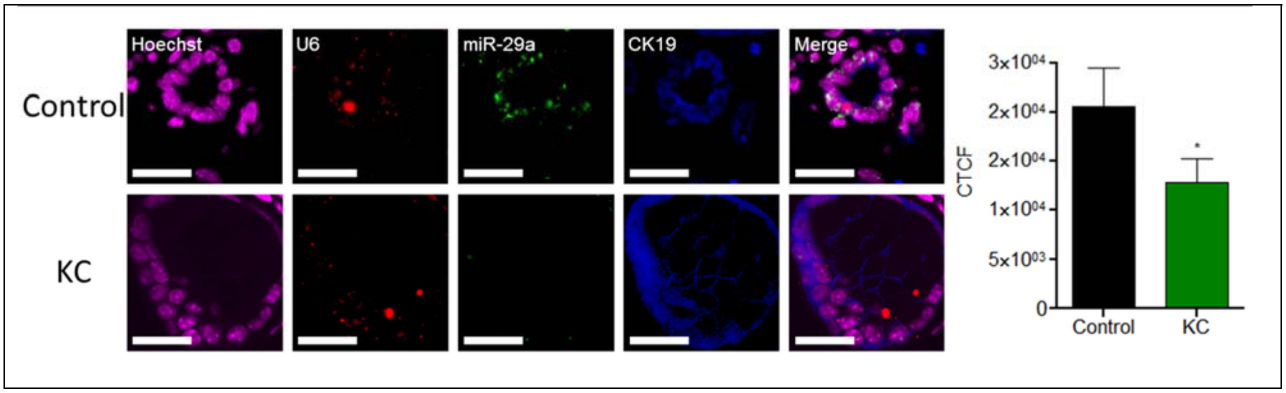

Supp Figure 8: miR-29a is decreased in CK19-positive epithelial cells in KC mice.
In situ hybridization of miR-29a in pancreata of control mice (C57BL/6) and KC mice. FFPE pancreatic tissue sections collected from 9-10 month old C57BL/6 control or KC mice (n=3/group/time point) were subjected to miR-29a *in situ* hybridization and images were generated using tyramide substrate amplification technique. Corrected total cell fluorescence (CTCF) of miR-29a was calculated for each animal by averaging six or more randomly selected CK19-positive epithelial cells using ImageJ analysis. Data represents the mean $\pm$ SEM; n=3; statistics generated by t-test, *p<0.05. Representative images are presented as a single channel, or merged (scale bar is 5µm, 20X magnification). Hoechst Nuclear stain (magenta), U6 (red), miR-29a (green), and CK-19 Epithelial stain (blue).

Supplementary Figure 9

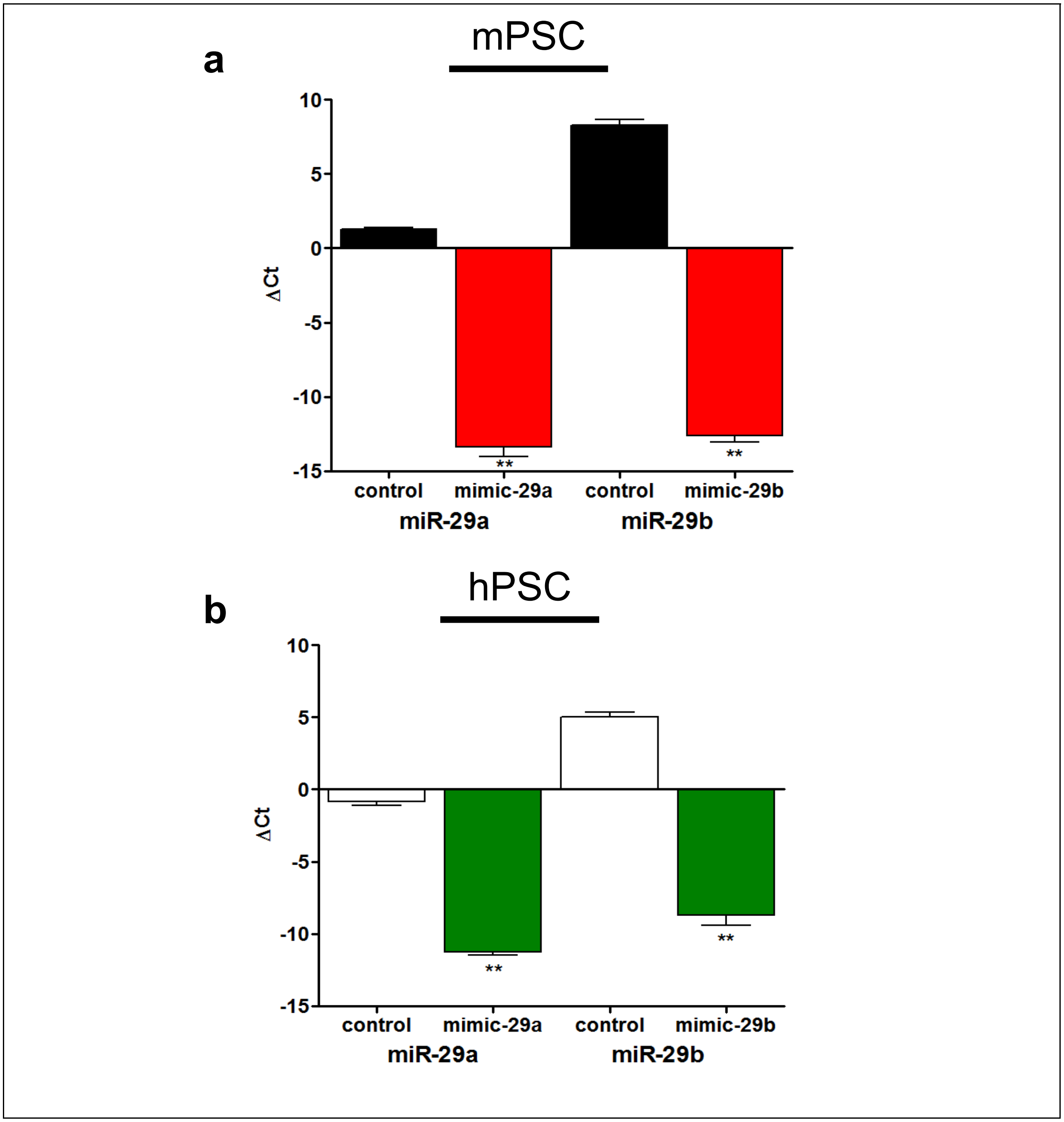

Supp Figure 9: Mouse and human PSCs transfected with miR-29 mimics have increased miR-29a and miR-29b expression. mPSCs (**a**) and hPSCs (**b**) were transfected with 25nM control, miR-29a, or miR-29b mimics. 24 hours post-transfection, RNA was extracted and miR-29a and miR-29b expression levels were quantified by qPCR using U6 snRNA as an internal control. Data is presented as mean ΔCT values ± SEM; n=3, statistics calculated using student t-test, **p<0.01.

Supplementary Figure 10

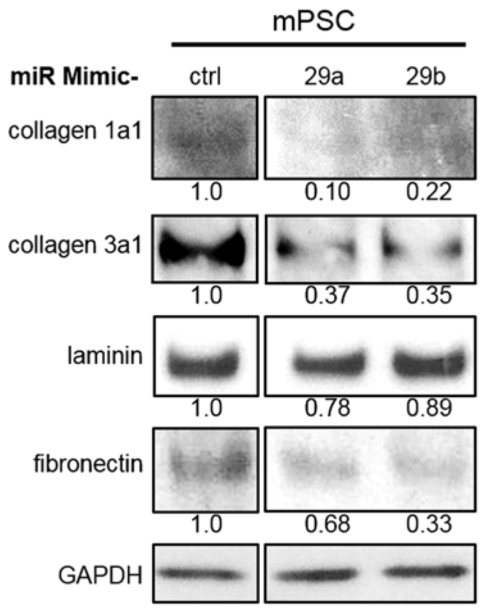

Supp Figure 10: Ectopic expression of miR-29 mimics reduce ECM components in TGF-β1 activated mouse PSCs. Mouse PSCs were serum starved for 24 hours and activated with 10ng/ml TGF-β1. 48 hours post-treatment, PSCs were transfected with miR-mimics (29a, 29b, or control) for 24 hours, and total proteins isolated. Western blot analysis was performed to determine the expression levels of ECM proteins (collagen 1a1, collagen 3a1, laminin gamma-1, and fibronectin) using GAPDH as a loading control. Relative quantification of band intensities, normalized to loading controls are shown below respective blots.

Supplementary Figure 11

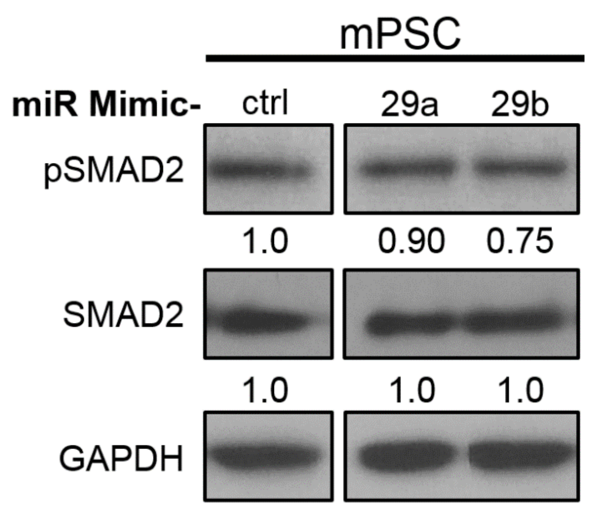

Supp Figure 11: miR-29 suppresses SMAD2 activation in mouse PSCs. mPSCs were transfected with 25nM miR-mimics, miR-29a (29a), miR-29b (29b), or mimic control (ctrl). Cells were then serum starved for 24 hours and activated with 10ng/ml TGF-β1 for 24 hours. Western blot analysis of pSMAD2 and SMAD2 was performed. Relative quantity was measured using GAPDH as a loading control and are shown below respective blots.

Kwon et al.

Supplementary Figure 12

a

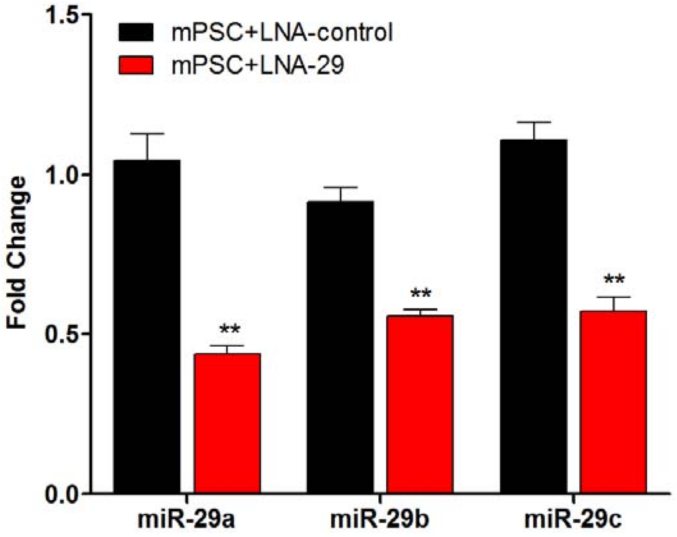

b

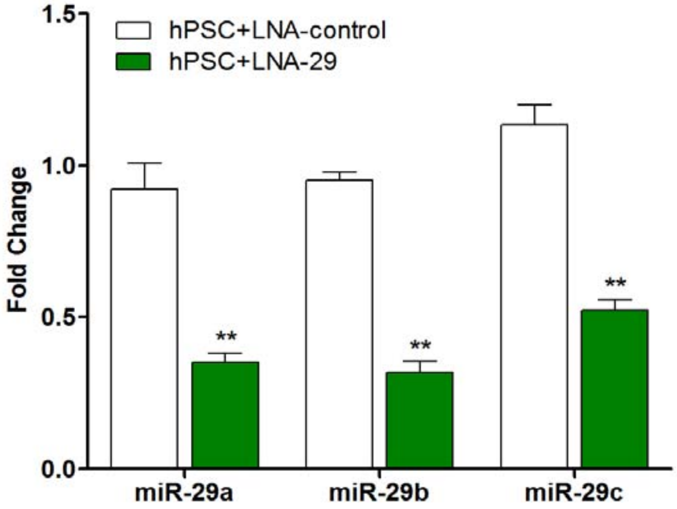

Supp Figure 12: LNA-miR-29 efficiently knockdown all endogenous miR-29 family members (miR-29a, miR-29b, and miR-29c) in mouse and human PSCs. mPSCs (**a**) and hPSCs (**b**) were transfected with 25nM LNA-control or LNA-miR-29 (LNA-29). 24 hours post-transfection, total RNA was isolated and miR-29 expression was quantified using qPCR analysis. Data is presented as mean ± SEM; n=3, statistics generated by student's t-test, **p<0.01.

Supplementary Figure 13

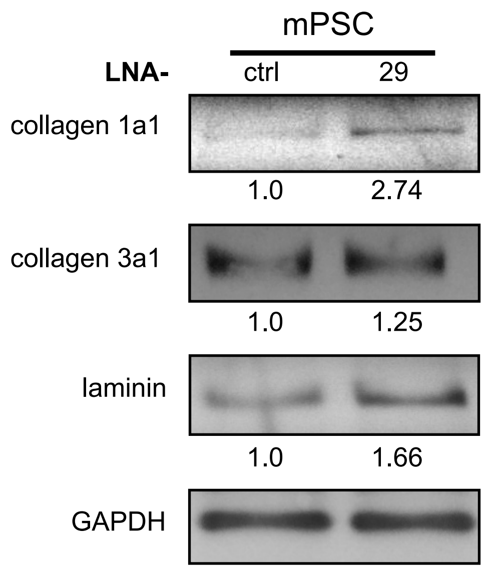

Supp Figure 13: miR-29 knockdown increases direct miR-29 ECM target proteins in mouse PSCs. mPSCs were starved for 24 hours, activated with 10ng/ml TGF-β1 for 24 hours, and transfected with LNA-control (ctrl) or LNA-29 (29). 24 hours post-transfection, total proteins were harvested to examine ECM proteins, collagen 1a1, collagen 3a1, and laminin gamma-1 (laminin) by western blot analysis using GAPDH as loading control. Relative quantification of band intensities normalized to loading control and are shown below respective blots.

Supplementary Figure 14

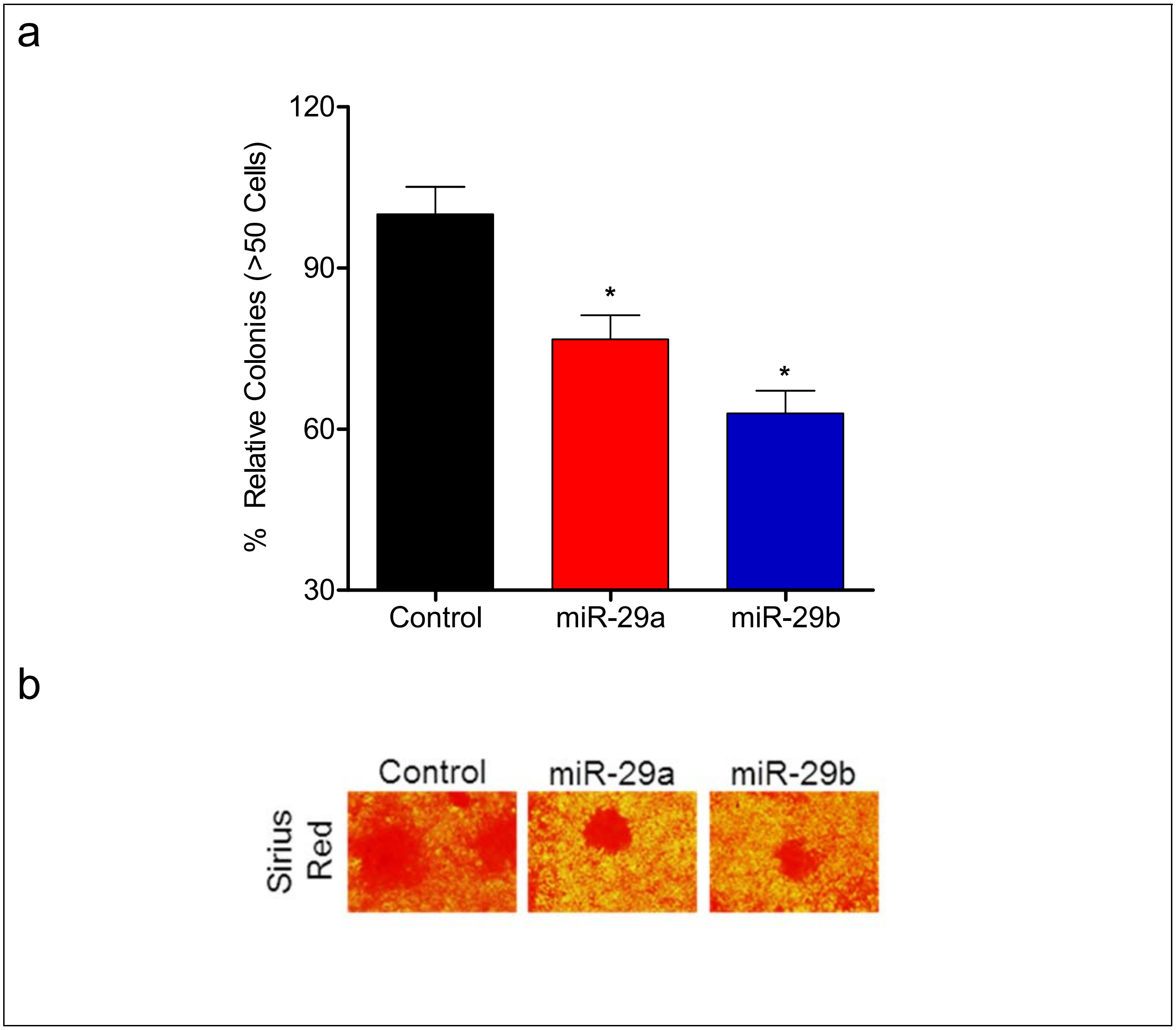

Supp Figure 14: Ectopic expression of miR-29 in PSCs causes reduced cancer colony formation and stromal deposition in direct co-cultures. (**a**) mPSCs transfected with mimic control, miR-29a, or miR-29b were co-seeded in 6-well plates with MIA PaCa2 cancer cells and cultured at 37°C for 10 days. Cancer colonies were stained with crystal violet and counted. Data is presented as relative percentage of cancer colonies normalized to control; mean $\pm$ SEM; n=3, statistics generated by student's t-test, *$p<0.05$.

(**b**) Effect of miR-29 overexpression in PSCs on stromal accumulation in direct co-cultures. Mouse PSCs transfected with mimic control, miR-29a, or miR-29b, were co-seeded in 6-well plates with MIA PaCa-2 cancer cell lines, cultured at 37° for 10 days, fixed, and subjected to Sirius Red staining. Representative images of co-cultures stained with Sirius Red are shown. All experiments were repeated 3-4 times and representative data is presented.

Supplementary Figure 15

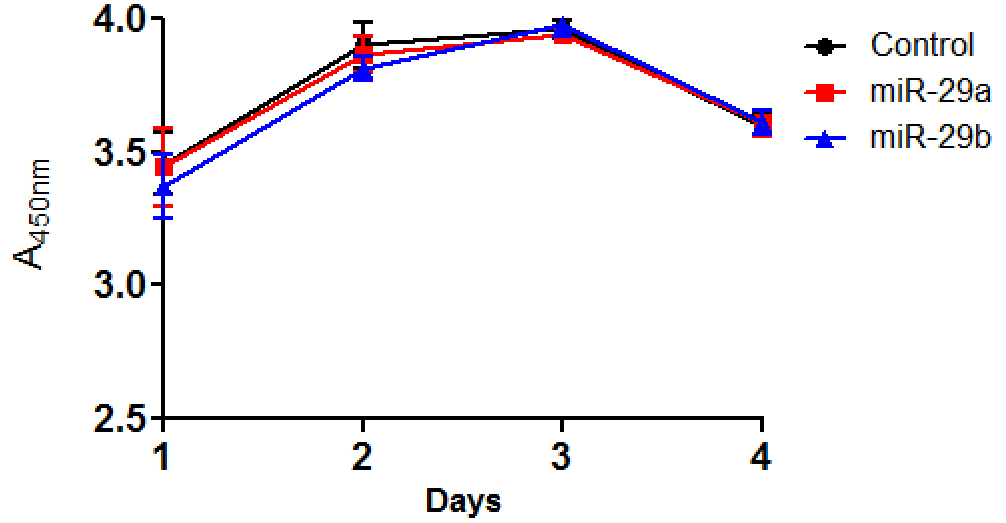

Supp Figure 15: Ectopic miR-29 expression does not affect PSC viability. PSCs were plated into 96-well plates, serum starved 24 hours, treated with 10ng/ml TGF-β1 for 24 hours, and then transfected with miR-control, miR-29a, or miR-29b. Cell viability was measured at 24, 48, 72, and 96 hours using the Cell Counting Kit-8 assay and absorbance was measured at 450nm. Data is presented as average relative absorbance ± SEM; n=4-8, statistics generated by student's t-test, but were not significant.

Supplementary Figure 16

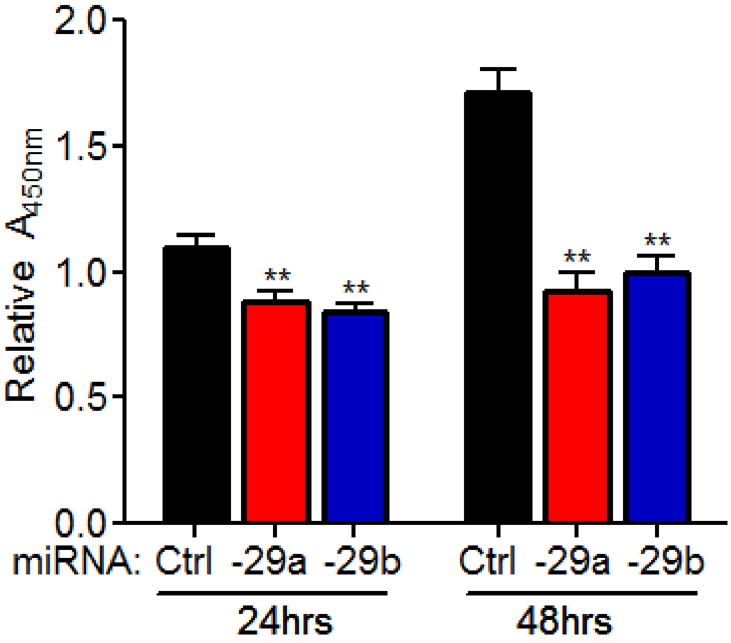

Supp Figure 16: Conditioned media of PSCs expressing miR-29 decreases pancreatic cell viability. Conditioned media from PSCs transfected with control, miR-29a, or miR-29b mimics was applied to MIA PaCa-2 cells in 96-well plates and viability was measured at 24 and 48 hours post-treatment using Cell Counting Kit-8 assay and absorbance was measured at 450nm. Data is normalized to MIA PaCa-2 cells treated with non-conditioned media. Average relative absorbance is presented as mean ± SEM; n=6-8, statistics generated by student's t-test, *p<0.05.

Supplementary Figure 17

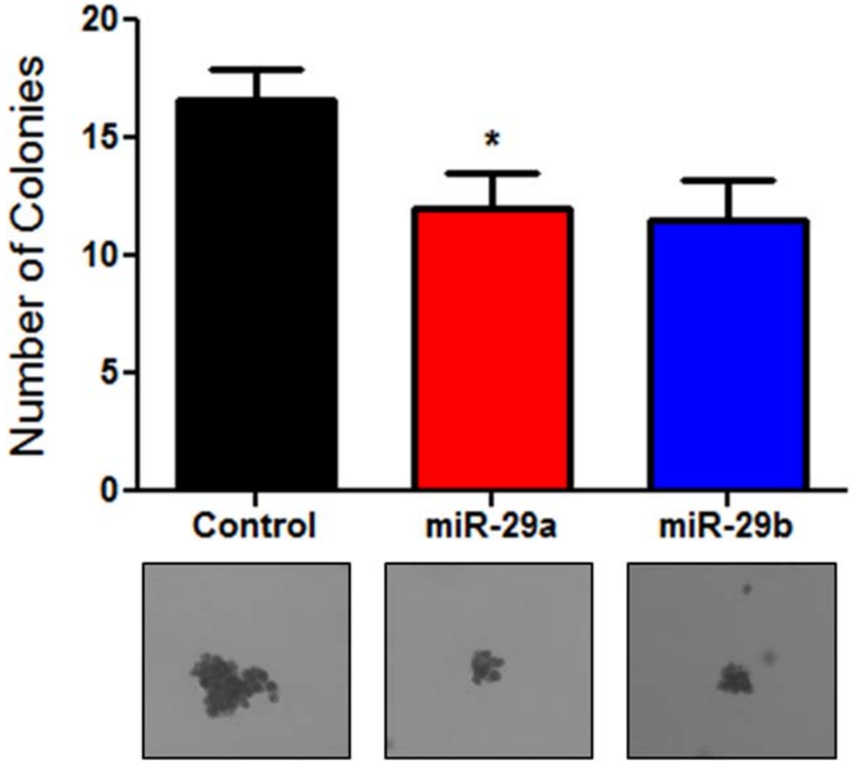

Supp Figure 17: miR-29 decreases the effect of PSCs on anchorage independent growth of pancreatic cancer cells. mPSC transfected with mimic control, miR-29a, or miR-29b were co-seeded in soft agar assay in a 6-well plate with pancreatic cancer cells (MIA PaCa-2) and were cultured at 37°C for 7 days. 500ul of medium was supplemented every other day. Following 7 days, cells were stained with crystal violet. Growth was examined microscopically and representative images are shown. Number of colonies per well is presented as mean $\pm$ SEM; n=6, statistics generated by student's t-test, *p<0.05.

Supplementary Table 1

Patient Data

	**Normal**	**PDAC**
Pancreas		
Normal	10	0
Tumor	0	15
Sex		
Male	5	9
Female	5	6
Tumor Stage		
T0	n/a	0
T1	n/a	1
T2	n/a	1
T3	n/a	13
Tumor Grade (differentiation)		
Well	n/a	1
Moderate	n/a	10
Poor	n/a	4
Lymph Node		
Positive	1	12
Negative	3	3
Not recorded	6	0
Age		
At procedure (average)	65.2	63.7
Range	38-77	42-83
Stroma		
35-50%	n/a	10
55-80%	n/a	5

Source: Indiana University Simon Cancer Center Tissue Bank and Department of Pathology

Supplementary Table 2

Sample type	Source	Sample ID#	% of stroma	Age	Sex	Ethnicity	Tumor location in pancreas- Head/Tail	Stage	Lymph Node- Positive/negative	Lymphovascular invasion (LVI)- positive/ negative	Perineural invasion (PNI)- positive/ negative	pTumor size (T0-T5)	Tumor Grade cannot be assessed (GX) Well (G1), Moderately (G2), or Poorly (G3) differentiated	Margins negative and positive
Normal	IU Pathology	N-01		73	F	white	Head		Negative		Positive			
Normal	IU Pathology	N-02		68	M	white	Head		positive (1/9)					negative
Normal	IU Pathology	N-03		38	F	white	not specified							
Normal	IU Pathology	N-04		55	F	white	not specified							
Normal	IU Pathology	N-05		70	M	white	not specified		Negative					negative
Normal	IU Pathology	N-06		73	M	white	Head		Negative					
Normal	IUSCC	N-07		74	F	non hispanic	not specified							
Normal	IUSCC	N-08		63	M	non hispanic	not specified							
Normal	IUSCC	N-09		61	F	non hispanic	not specified							
Normal	IUSCC	N-10		77	M	non hispanic	not specified							
Tumor	IU Pathology	PDAC-01	50	64	M	white	head	pT3, N1, MX	positive (10/19)	positive	Positive	T3	Moderatly (G2)	negative
Tumor	IU Pathology	PDAC-02	80	66	M	white	Head	pT3, pN1, pMX	positive (9/11)	positive	Positive	T3	Poorly (G3)	positive
Tumor	IU Pathology	PDAC-03	70	67	M	white	Head	pT3, pN1, pMX	positive (8/13)	positive	Positive	T3	Moderatly (G2)	positive
Tumor	IU Pathology	PDAC-04	50	71	M	white	Head	pT3, N1, MX	Positive (1/12)	Negative	Positive	T3	Moderatly (G2)	negative
Tumor	IU Pathology	PDAC-05	65	68	M	white	Head	pT3, pN1, pMX	positive (9/21)	positive	Positive	T3	Well to Moderatly	positive
Tumor	IU Pathology	PDAC-06	70	69	F	black	Head	pT3, N0, MX	negative	Negative	Positive	T3	Poorly (G3)	negative
Tumor	IUSCC	PDAC-07	60	75	F	non hispanic	Head	T2, N1, MX	positive		Positive	T2	Moderatly (G2)	
Tumor	IUSCC	PDAC-08	40	54	M	non hispanic	Head	T3, N1, MX	positive		Positive	T3	Moderatly (G2)	
Tumor	IUSCC	PDAC-09	40	60	F	non hispanic	Head	T3, N1, Mx	positive		Positive	T3	Moderatly (G2)	
Tumor	IUSCC	PDAC-10	35	57	M	non hispanic	Tail	T3, N1, MX	positive	positive	Positive	T3	Moderatly (G2)	
Tumor	IUSCC	PDAC-11	40	73	F	non hispanic	Head	T1, N0, MX	negative		Positive	T1	Poorly (G3)	
Tumor	IUSCC	PDAC-12	50	83	F	non hispanic	Head	T3, N0, MX	negative		Positive	T3	Moderatly (G2)	
Tumor	IUSCC	PDAC-13	50	53	F	non hispanic	Head	T3, N1, MX		positive	Positive	T3	Moderatly (G2)	
Tumor	IUSCC	PDAC-14	50	42	M	non hispanic	Tail	T3, N1, MX		Negative	Positive	T3	Moderatly (G2)	
Tumor	IUSCC	PDAC-15	50	53	M	non hispanic	Tail	T3, N1, MX	positive	negative	Postive	T3	Poorly (G3)	

Source: Indiana University Simon Cancer Center Tissue Bank and Department of Pathology
